# TGF-β regulates Sca-1 expression and plasticity of pre-neoplastic mammary epithelial stem cells

**DOI:** 10.1038/s41598-020-67827-4

**Published:** 2020-07-09

**Authors:** Ján Remšík, Markéta Pícková, Ondřej Vacek, Radek Fedr, Lucia Binó, Aleš Hampl, Karel Souček

**Affiliations:** 1 Institute of Biophysics of the Czech Academy of Sciences, Královopolská 135, 612 65 Brno, Czech Republic; 2 Center of Biomolecular and Cellular Engineering, International Clinical Research Center, St. Anne’s University Hospital Brno, Pekařská 53, 656 91 Brno, Czech Republic; 3 Department of Experimental Biology, Faculty of Science, Masaryk University, Kamenice 5, 625 00 Brno, Czech Republic; 4 Department of Histology and Embryology, Faculty of Medicine, Masaryk University, Kamenice 5, 625 00 Brno, Czech Republic; 5 Present Address: Human Oncology and Pathogenesis Program, Memorial Sloan Kettering Cancer Center, New York, NY 10065 USA

**Keywords:** Breast cancer, Cancer stem cells

## Abstract

The epithelial-mesenchymal plasticity, in tight association with stemness, contributes to the mammary gland homeostasis, evolution of early neoplastic lesions and cancer dissemination. Focused on cell surfaceome, we used mouse models of pre-neoplastic mammary epithelial and cancer stem cells to reveal the connection between cell surface markers and distinct cell phenotypes. We mechanistically dissected the TGF-β family-driven regulation of Sca-1, one of the most commonly used adult stem cell markers. We further provided evidence that TGF-β disrupts the lineage commitment and promotes the accumulation of tumor-initiating cells in pre-neoplastic cells.

## Introduction

The mechanisms of cellular plasticity, de-differentiation and trans-differentiation, are in the physiological context essential for the tissue regeneration and maintenance of homeostasis ^1^. Although the postnatal mammary gland contains mostly unipotent progenitors contributing to stage-specific events, lineage tracing and single-cell studies showed evidence that the mammary stem cell compartment is heterogeneous and that the various mechanisms of bi-directional plasticity appear during branching morphogenesis, involution and evolution of breast cancer^2,3^. The terminal differentiation of mammary epithelial cells is contributed by paracrine signals and epigenetic regulation, and their abrogation leads to de-differentiation^4^. Importantly, the de-differentiation is often associated with the somatic reprogramming, and linked to the tumor-initiating capabilities and oncogenic transformation^5^.

Stem cell antigen-1, known as Sca-1, is one of the most commonly used markers of normal mouse stem cells and progenitors^6,7^ or cells with increased tumorigenic potential^8,9^. Despite that, the molecular mechanisms associated with cellular plasticity and responsible for Sca-1 ‘off-and-on’ switching remain largely unidentified^10^. Sca-1 is a glycosylphosphatidylinositol-anchored surface protein and a member of LY6 family, interacting with the TGF-β receptors and TGF-β ligands to modulate the downstream signaling in multiple organs^11,12^. Its contribution to the maintenance of stem and progenitor transcriptional programs is unknown. Identification of mechanisms that collaborates in its regulation may help to answer several important questions in the biology of normal and cancer stem cells.

In this report, we used HER2 over-expressing mouse mammary epithelial cancer cells (MMC) and their mesenchymal counterparts in which the loss of HER2 was driven by massive immunoediting in vivo (thus HER2-negative or “antigen-negative variants”, ANVs^13^). We demonstrated that Sca-1 marked cancer cells with stem-like characteristics. Similarly, in the model of pre-neoplastic mammary epithelial cells Comma-Dβ, Sca-1 was expressed in the basal-like subpopulation enriched in mammary progenitors. The transient exposure of these cells to TGF-β resulted in the loss of Sca-1 and selection of or enrichment in cells with increased tumorigenic potential. While the endogenous TGF-β signaling repressed Sca-1 through Smad2/3/4, the inhibition of Sca-1 expression upon exogenous TGF-β stimuli was Smad2/3-independent. In summary, we showed that TGF-β signaling regulates Sca-1 expression, tumorigenicity and plasticity of mammary epithelial and cancer stem cells.

## Materials and methods

All methods were performed in accordance with the relevant guidelines and regulations, as stated in relevant sections below.

### Cell lines, cell culture, and transfections

Her2/*neu*-derived cell lines (MMC, ANV2, ANV5^13^) were cultured in RPMI 1640 (Thermo Fisher Scientific/TFS, Gibco, Prague, Czech Republic) supplemented with 20% fetal bovine serum (Gibco), 1% natrium pyruvate (Sigma-Aldrich, Prague, Czech Republic), 1% penicillin/streptomycin (Biosera, Nuaille, France). Comma-Dβ cell line was cultured in DMEM/F12 (1:1, Gibco) supplemented with 2% fetal bovine serum, human recombinant insulin (10 μg/mL; Sigma-Aldrich), murine EGF (5 ng/mL; Sigma-Aldrich) and 1% penicillin/streptomycin. HEK293 (CRL-1573, ATCC) cells were cultured in low glucose DMEM (Gibco), supplemented with 10% fetal bovine serum and 1% penicillin/streptomycin. All transfections were performed using Lipofectamine 3000 (Invitrogen), if not stated otherwise, as recommended by the manufacturer. Endoribonuclease-prepared short interfering RNAs (siRNA) were acquired from Sigma-Aldrich (Smad2 cat. no. EMU022831-20UG, Smad3 cat. no. EMU014271-20UG, Smad4 cat. no. EMU016021; siRNA targeting EGFP cat. no. EHUEGFP was used as a scrambled control). Sca-1 over-expression (Sca-3T) and empty vectors bearing neomycin resistance were described in^14^ and kindly provided by Drs. Karen Westerman and Claudio Perez (Birgham and Women’s Hospital, Boston, MA, USA). Overexpression was validated with flow cytometry. All cell lines were routinely tested for mycoplasma contamination using PCR. The AmpFLSTR Identifiler PCR Amplification Kit (Applied Biosystems, TFS) was used to verify the origin of HEK293 cell line.

### Cytokines, small molecule kinase inhibitors, and receptor chimeras

Recombinant human (rh-)TGF-β1 (Millipore, Burlington, MA, United States), rh-BMP2, rh-BMP4, rh-BMP7, rh-GDF15 and rh-Activin A (all from Peprotech, Rocky Hill, NJ, United States) were dissolved in 0.1% BSA containing PBS and stored as recommended. Galunisertib, LDN-193189, LY2109761 (ApexBio, Houston, TX, United States), dorsomorphin (Biomol, Hamburg, Germany), SIS3, TGFBR1 kinase inhibitor IV (Calbiochem, San Diego, CA, United States), decitabine, K02288 (Selleckchem, Houston, TX, United States), SB431542 hydrate, RepSox (Sigma-Aldrich), GW788388, DMH-1 (Tocris, Bristol, United Kingdom) were dissolved in DMSO (Sigma-Aldrich) and stored according to manufacturer’s recommendations. Recombinant human Type II TGF-β receptor chimeras ACVR2A-Fc, ACVR2B-Fc, BMPR2-Fc, TGFBR2-Fc, and IgG-Fc were purchased from R&D (Minneapolis, MN, United States) and dissolved in 0.1% BSA containing PBS and stored as recommended.

### Animal experiments

The colony of severe combined immunodeficient animals (Crl:SHO-*Prkdc*^*scid*^*Hr*^*Hr*^) was acquired from Charles River (Sulzfeld, Germany) and maintained according to the ARRIVE guidelines. For orthotopic mammary fat pad injections, suspension of 7.5 × 10^5^ (Comma-Dβ), 5 × 10^4^ (ANV2), or 2.5 × 10^5^ (MMC) cells in 50 μL PBS were injected into fourth mammary gland of 6 weeks old females. Tumor dimensions were measured with the calibrated digital caliper (VWR, Radnor, PA, United States). Mice were sacrificed when the tumor size reached the end-point specified in the animal protocol, the date was recorded and use for Mantel-Cox logrank analysis (overall survival). Experiments were terminated twelve weeks after tumor cell injection. For implantations into cleared mammary fat pad, 1 × 10^5^ cells in 50 μL PBS were implanted into fourth mammary gland of 6 weeks old females, with mammary fat pad cleared at the age of 3 weeks using sparing procedure, as described previously^15^. Animal experiments were approved by the Academy of Sciences of the Czech Republic (AVCR 2015/13); supervised by the local ethical committee and performed by the certified individuals (JR, OV, KS).

### Antibody-based cell surface screening, and data analysis

The high-throughput surface profiling and data analysis were performed essentially as described in^16^ with minor modifications specified below, as different cell lines and kit components were used in this study. Epithelial cell lines were incubated in 1.35 mM EDTA solution in PBS prior to trypsinization to allow the non-enzymatic weakening of the cell junctions prior to trypsinization (10 min for MMC and cE2 cells). Mesenchymal cell lines (ANV2, E2, RM-1 and UGSM-2 cells) were only briefly washed with EDTA solution, as prolonged incubation would lead to complete cell detachment. Cell lines were then harvested with mild 0.05% trypsin/0.02% EDTA solution (5 min at 37 °C, GE Healthcare, Little Chalfont, United Kingdom, cat. L15-004). Next, the cell suspensions were processed as described previously, except that 4.2 × 10^7^ cells from each, re-counted cell line were then pooled. The pool of cells was washed with PBS and stained with LIVE/DEAD Green Fixable Dead Cell Stain diluted 1:1,000 in PBS for 15 min at 4 °C (1 mL staining solution per 1 × 10^7^ cells; Molecular Probes, TSF). Next, cells were washed, resuspended in 25.2 mL Cell Stain buffer and filtered via 70 μm cell strainer to remove large cell aggregates. Filtered cell suspension (75 μL/well, equal to pool of 0.75 × 10^6^ cells/well) was dispensed into LEGENDScreen Mouse Phycoerythrin (PE) Kit 96 well plates (cat. 700005; Biolegend, San Diego, California, United States), each well was already containing 25 μL of single, validated and pre-titrated antibody conjugated with PE. Plates were reconstituted with deionized water, and cells were then stained and fixed as recommended by the manufacturer. Data acquisition and analysis were described previously. Since we further focused on mammary gland biology, results from RM-1^17^, UGSM-2^18^, cE2 and E2^19^ prostate-derived cell lines were not presented in this manuscript. However, they are included in the Supplementary Table 1 and 2.

### Instrumentation, flow cytometry analysis, and cell sorting

Flow cytometry was performed as described in^16^. The acquisition was performed on FACSVerse (BD Biosciences, Franklin Lakes, NJ, United States), Attune Acoustic Focusing Cytometer (Applied Biosciences, TFS), or SP-6800 Spectral Analyzer (Sony Biotechnology, Tokyo, Japan) and re-analyzed using FlowJo Single Cell Analysis Software (v10.0.7; TreeStar, Ashland, OR, United States). Cell aggregates, doublets, and debris were excluded from analysis based on a dual-parameter dot plot in which the pulse ratio (signal height/y-axis versus signal area/x-axis) was displayed. Dead cells were excluded from analysis by staining with propidium iodide (Sigma-Aldrich) or LIVE/DEAD Fixable Dead Cell Stain (various fluorescence reactive dyes, Molecular Probes, TFS). Viable single cells were sorted using FACSAria II Sorp system (BD). For all sortings, a 100-μm nozzle (20 psi) was used, and post-sorting purity was determined immediately after sorting. In experiments involving seeding of sorted cells into medium with or without TGF-β1, cells were cultured for 96 h to reach typical confluence (in regular experiments, cells were treated 24 h after seeding and cultured for another 72 h). This experiment was performed in two biological replicates and technical duplicates. Compensation values for multicolor analyses were calculated automatically in FlowJo from single-conjugate stained UltraComp eBeads (eBiosciences, TFS). All antibodies were titrated prior to use and used as determined by titration or as recommended. Dilution, clonality, conjugation and catalog numbers of the antibodies used for flow cytometry are listed in the Supplementary Table 3.

### Spheroid formation assay

Cell lines were harvested using PBS/EDTA and trypsin/EDTA to obtain single-cell suspensions, as described above. 1,000 cells in 200 μL of cell culture medium was seeded per each well of 96-well ultra-low attachment plates (Corning #7007). 150 μL of fresh medium was replenished after 48 h and images were taken 96 h after initial seeding using ImageXpress Micro XLS Widefield (Molecular Devices, Sunnyvale, California, United States) equipped with the 4 × objective. Spheroid size was determined in MetaXpress (v5.1, Molecular Devices) using an automatic pipeline as follows: objects were identified using Gaussian filter 2, inverted images were then processed with Filter Mask (threshold for perimeter was set to 800 and for circularity 0.15), and quantified.

### ABC transporter and ALDH activity assays

Cell lines were harvested using PBS/EDTA and trypsin/EDTA to obtain single-cell suspensions, as described above. For determination of ABC transporter activity, cell suspensions were incubated with JC-1 (final concentration 1 μM; Sigma-Aldrich, cat. T4069) or mitoxantrone (MTX, final concentration 15 μM; Sigma-Aldrich, cat. T6545) for 1 h at 37C in the dark. Fumitremorgin C (final concentration 10 μM; Sigma-Aldrich, cat. T9054) was used as a positive control for ABC transporter activity inhibition. ALDH activity was determined using Aldefluor Kit (StemCell, cat. 01700), as recommended. Samples, together with autofluorescence controls manipulated the same way were recorded with SP-6800 Spectral Analyzer (JC-1 and Aldefluor 488 nm excitation laser, MTX 640 nm excitation laser).

### Analysis of cell cycle, EdU incorporation assay, apoptosis, and cell death

For multiparametric analysis of surface marker expression and proliferation, cells were treated with 10 μM EdU two hours prior to harvesting. Cells were then trypsinized, stained for surface markers, fixed in 4% formaldehyde, permeabilized with 0.15% Triton X-100. Click-iT reaction was performed with Click-iT EdU Alexa Fluor 488 Flow Cytometry Kit, and cell cycle was analyzed with FxCycle Violet (Molecular Probes, TFS) according to manufacturer’s recommendation. For analysis of cell death, cells (including the floating fraction) were collected, stained for surface Sca-1 as described above, followed by staining with Annexin V-FITC conjugate (#ANXV-FT100, Apronex, Prague, Czech Republic) in annexin V staining buffer, as recommended. Propidium iodide was added to the suspension one minute before analysis. Analysis was performed on SP-6800 Spectral Analyzer.

### RNA isolation, reverse transcription, and qPCR analysis

Total RNA from either cell culture or freshly sorted cells was extracted using High Pure RNA Isolation Kit (Roche) and quantified on BioSpectrometer (Eppendorf, Hamburg, Germany). cDNA synthesis was performed with 0.2–2 μg of total RNA using High-Capacity RNA-to-cDNA Kit (Applied Biosystems, TFS). mRNA levels were measured with gene-specific primers using Roche LightCycler 480 master mix, probes and thermocycler system (Roche, Basel, Switzerland) and relative expression levels were normalized to the reference gene *Tbp*. Gene expression assays used for qPCR analysis are listed in the Supplementary Table 4.

### Western blot analysis

Western blot analyses were performed as described previously^20^, except that selected blots were visualized with ChemiDoc XRS Imaging System (Bio-Rad, Hercules, CA, United States). The dilution, clone and catalog numbers of the antibodies used are listed in the Supplementary Table 5.

### Luciferase reporter assays

100,000 of HEK293 cells were plated per well in 48-well plates. 24 h after seeding, 100 ng *Firefly* luciferase reporter was co-transfected with 20 ng pRL-TK *Renilla* luciferase vector (Promega, Madison, WI, United States) per well, using ViaFect (Promega). 24 h after transfection, cells were starved for 4 h and exposed to experimental treatment for 2 h. Plates were then washed, lysed in 1 × Passive Lysis Buffer (Promega) and frozen at − 20 °C to enhance the disruption of cell membranes. Luciferase activity in cell lysates was measured using Dual-Luciferase Reporter Assay System (Promega), the activity of *Firefly* luciferase was normalized to that of *Renilla*. Control samples were analyzed in technical duplicates, and experimental samples were analyzed in technical quadruplicates.

### Enzyme-linked immunosorbent assay

Levels of total and native TGF-β1 were quantified using Human/Mouse TGF beta 1 Ready-SET-Go! ELISA Set (eBioscience, TFS) from 24 h serum-free culture supernatants following manufacturer’s instructions. Levels of total TGF-β1 were determined from undiluted culture supernatants (100 μL/well) treated for 15 min with 1 M HCl (20 μL/well), followed by neutralization with 1 M NaOH (20 μL/well) and normalized by the dilution factor ‘1.4’. Levels of active TGF-β1 were determined directly in the supernatants (100 μL/well), without acidification and neutralization. Each sample was loaded in technical duplicate.

### Statistical analysis, software, and repeatability of experiments

Statistical analyses were carried out by two-tailed Student’s t-test in Prism (v6, GraphPad, La Jolla, CA, United States) if not stated otherwise. The protein sequences were acquired from UniProt (https://www.uniprot.org), and the LY6 family phylogenetic tree was generated with Phylogeny.fr^21^ and further re-drawn in Graphic (Picta). No statistical methods were used to predetermine sample size. The experiments were not randomized, and the investigators were not blinded to allocation during experiments and outcome assessment. Detailed information about replicates is described in figure legends. Raw data were recorded and/or processed and/or visualized using FlowJo (v10.0.7, TreeStar), GENE-E (Broad Institute), LightCycler 480 Software (v1.5, Roche), Image Lab Software (v.5.2.1 for Mac, Bio-Rad) and Prism (v6 and v7, GraphPad).

## Results

### Sca-1 is differentially expressed on the cell surface of mammary cancer stem cells

**Figure 1:**
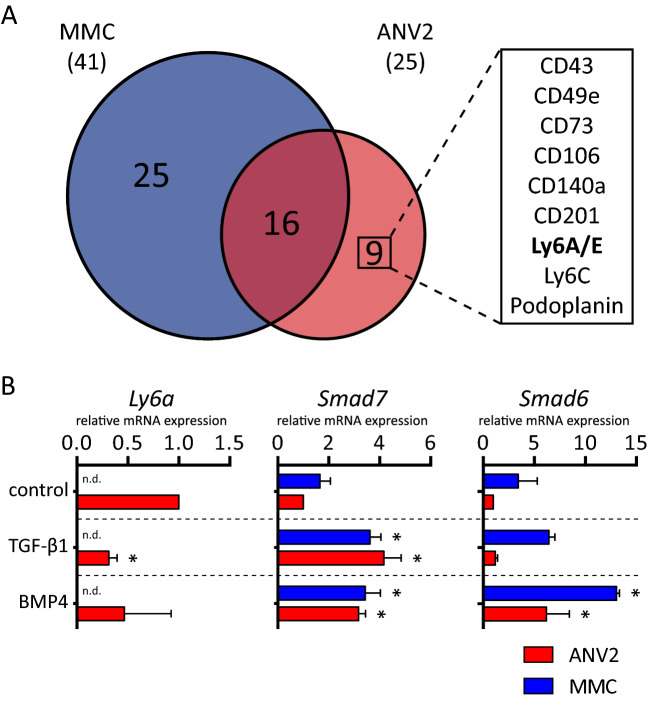
Stem cell phenotype associates with distinct surface signature in ANV2 cells. **(A)** Venn diagram shows overlap of surface antigens upregulated on surface of epithelial cell line MMC and its mesenchymal counterpart ANV2. Using high-throughput surface profiling we identified nine differentially expressed mesenchymal surface antigens, listed in the box. **(B)** Plots show changes in Sca-1 gene expression (*Ly6a*) in MMC and ANV2 cells upon 24 h exposure to vehicle (PBS with 0.1% BSA), TGF-β1 (10 ng/mL), and BMP4 (100 ng/mL). Results are from three independent experiments and are presented as mean ± SD (*P < 0.05, *t* test). *Smad6* and *Smad7* serve as positive controls of pathway activation.

Acquisition of cancer stem cell phenotype is often accompanied by a dramatic and complex remodeling of the cell membrane and other cellular components. To gain deeper insight into the changes of cell surface antigens accompanying such epithelial-mesenchymal plasticity, we performed multiplexed, high-throughput cell surface profiling (Fig. S1A-B and Supplementary Table 1 and 2, for technical details see^16^). We identified nine surface antigens that were upregulated on the cell surface of ANV2 cells (Fig. 1A). These HER2 antigen-negative variants (ANV) are exhibiting stem-like properties and were derived from spontaneously relapsed and immune-edited tumors after injection of purely epithelial murine mammary cancer cells (MMC) into the syngeneic hosts (Fig. S2A-E and^13,22^). Stem cell antigen-1 (Sca-1 or Ly6A/E) was present exclusively on the surface of ANV2 cells. In order to verify the observed differences in the cytokine-induced model of EMT, we exposed MMC and ANV2 cells to several TGF-β family ligands. To our surprise, TGF-β1 mediated down-regulation of Sca-1 expression in ANV2 cells (Fig. 1B). In our experimental settings, Sca-1 marked mesenchymal cancer cells with stem-like properties and its expression was down-regulated in the presence of TGF-β1.

**Figure 2:**
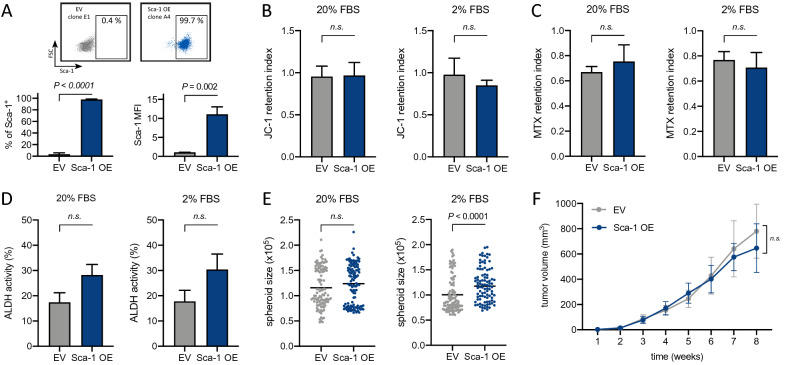
Sca-1 is not sufficient to induce stem-like phenotype in mammary epithelial cancer cells. **(A)** Representative dot plots and bar graphs show efficiency of constitutive Sca-1 overexpression in non-stem epithelial MMC cells (Sca-1 OE) and their empty vector controls (EV). Results are from four measurements from two independent clones *per* subline and are presented as mean ± SEM (*t* test). **(B)** Plots show capacity of Sca-1 OE and EV MMC cells to retain JC-1 as a proxy of ABC transporter activity in mitogen-high (20% FBS, standard cell culture) and mitogen-low conditions (2% FBS). Results are from four measurements from two independent clones *per* subline and are presented as mean ± SEM (*t* test). **(C)** Plots show capacity of Sca-1 OE and EV MMC cells to retain mitoxantrone as a proxy of ABC transporter activity in mitogen-high (20% FBS, standard cell culture) and mitogen-low conditions (2% FBS). Results are from four measurements from two independent clones *per* subline and are presented as mean ± SEM (*t* test). **(D)** Plots show percentage of Sca-1 OE and EV MMC cells exhibiting ALDH activity in mitogen-high (20% FBS, standard cell culture) and mitogen-low conditions (2% FBS). Results are from four measurements from two independent clones *per* subline and are presented as mean ± SEM (*t* test). **(E)** Scatter plots show spheroid size of MMC sublines as determined with spheroid formation assay. Results are from two independent experiments from two independent clones *per* subline (Mann–Whitney *u* test). (F) Plot shows tumor growth of Sca-1 OE (n = 18) and EV MMC cells (n = 15). Results are presented as mean ± SEM (Mann–Whitney *u* test).

Despite that Sca-1 is one of the most commonly used markers for adult murine stem cells, its contribution to stemness is not yet understood in many tissue types. We overexpressed Sca-1 in epithelial MMC cells that do not display stem-like properties and assessed their phenotype and behavior in vitro and in vivo. Cells over-expressing ectopic Sca-1 did not demonstrate increased ABC transporter or ALDH activity, elevated spheroid formation capacity under standard culture conditions (Fig. 2A–E), or enhanced tumor growth (Fig. 2F). Sca-1 itself is thus not sufficient to induce stem cell phenotype in mammary epithelial cancer cells.

### TGF-β affects the differentiation state of mammary epithelial cells

**Figure 3:**
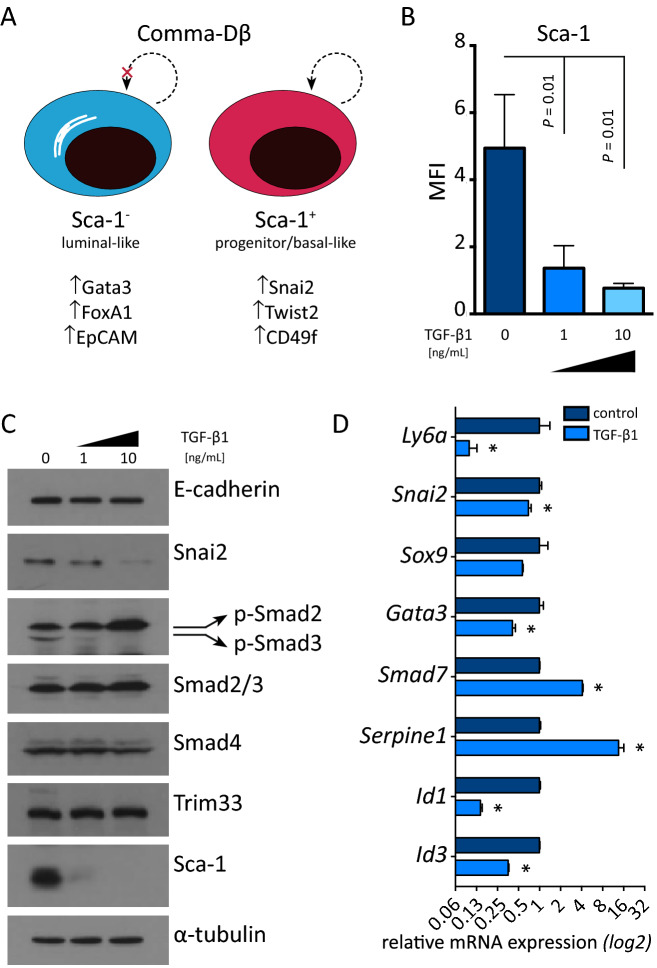
TGF-β signaling promotes Sca-1 down-regulation in mammary epithelial cells. **(A)** The scheme shows association of Sca-1^-^ subpopulation of Comma-Dβ with luminal phenotype and Sca-1^+^ subpopulation with basal phenotype, based on their gene expression pattern and surface profile. See also Supplementary Fig. 2. **(B)** The plot shows changes in surface Sca-1 expression in Comma-Dβ cells upon 72 h exposure to selected concentrations of TGF-β1, analyzed with flow cytometry. Results are from three independent experiments and are presented as mean ± SD, *t* test (MFI = median fluorescence index). **(C)** Representative western blots from three independent experiments show the expression levels of E-cadherin, Snai2/Slug, phospho-Smad2(Y465/467)/Smad3(Y423/425), total Smad2/3, Smad4, Trim33, Sca-1 and α-tubulin. Comma-Dβ cells were exposed to selected concentrations of TGF-β1 for 72 h. **(D)** The plot shows changes in gene expression of Sca-1 mRNA (*Ly6a*), regulators of basal phenotype *Sox9* and *Snai2*, regulator of luminal phenotype *Gata3* and TGF-β target genes *Smad7*, *Serpine1*, *Id1* and *Id3*, presented as mean ± SD (multiple* t* tests, * FDR *q* < 0.05). Comma-Dβ cells were exposed to 1 ng/mL TGF-β1 for 72 h. Overall gene expression change after TGF-β1 exposure *P* value = 0.0003 (two-way ANOVA).

We explored the effect of TGF-β1-mediated Sca-1 down-regulation in the context of pre-neoplastic mammary epithelial cells. The Comma-Dβ cell line is derived from the normal mammary gland of mid-pregnant mice and serves as a pre-neoplastic cell line model for studying mammary gland plasticity^23,24^. Comma-Dβ cells are known for their heterogeneous expression of Sca-1: Sca-1^+^ subpopulation is enriched in mammary progenitors^23^. We first extensively characterized both the Sca-1^−^ and Sca-1^+^ subpopulations of these cells, confirming that Sca-1^−^ fraction resembled the luminal-like mammary epithelial cells, while the Sca-1^+^ fraction showed increased expression of basal-like markers (*Snai2*, *Twist2*, Cd49f; Fig. 3A and S3A-B). Interestingly, the other members of the LY6 family – *Ly6c1*, *Ly6c2*, *Ly6e*—showed similar expression pattern to that of Sca-1 (Fig. S3A-D), suggesting their potential in co-maintenance of the progenitor functions and presence of multiple other subpopulations.

The Comma-Dβ cells responded to TGF-β1 by almost complete surface ablation of Sca-1, in a dose-dependent manner (Fig. 3B and S4A). We analyzed the TGF-β-induced expression changes in both subpopulations, as well as the effect of TGF-β on cell death and proliferation. Despite that TGF-β1-mediated loss of Sca-1 was not accompanied by the epithelial-to-mesenchymal transition (Fig. 3C and S3E), it resulted in a down-regulation of transcription factors Slug/Snai2^25^ and Gata3^26^, responsible for lineage commitment (Fig. 3D). Similar to TGF-β1, the bone morphogenetic proteins BMP4 and BMP7 also efficiently repressed Sca-1 expression (Fig. S4B-C). Pharmacologic inhibition of TGF-β, but not that of BMP, led to the increase in Sca-1 expression, independently of a pharmacophore (Fig. S4D-E). Simultaneous kinetic analysis of cell surface Sca-1, DNA synthesis and cell cycle progression further revealed that the complete loss of surface Sca-1 in response to TGF-β1 appeared after 72 h (Fig. S5A-B). The Sca-1^−^ and Sca-1^+^ subpopulations had distinct cell cycle profiles even without perturbation (while 44% of Sca-1^−^ cell is in G1/0 and 51% in G2/M; 61% of Sca-1^+^ cell is in G1/0 and 36% in G2/M). Modulation of the TGF-β pathway did not alter the cell cycle pattern and neither did induce apoptosis (Fig. S5C-D). Based on these results we conclude that the observed TGF-β1-induced phenotypic changes in Comma-Dβ cells are not the result of aberrant proliferation or induction of cell death.

### The short-term exposure of pre-neoplastic mammary epithelial cells to TGF-β enriches for tumor-initiating cells

**Figure 4:**
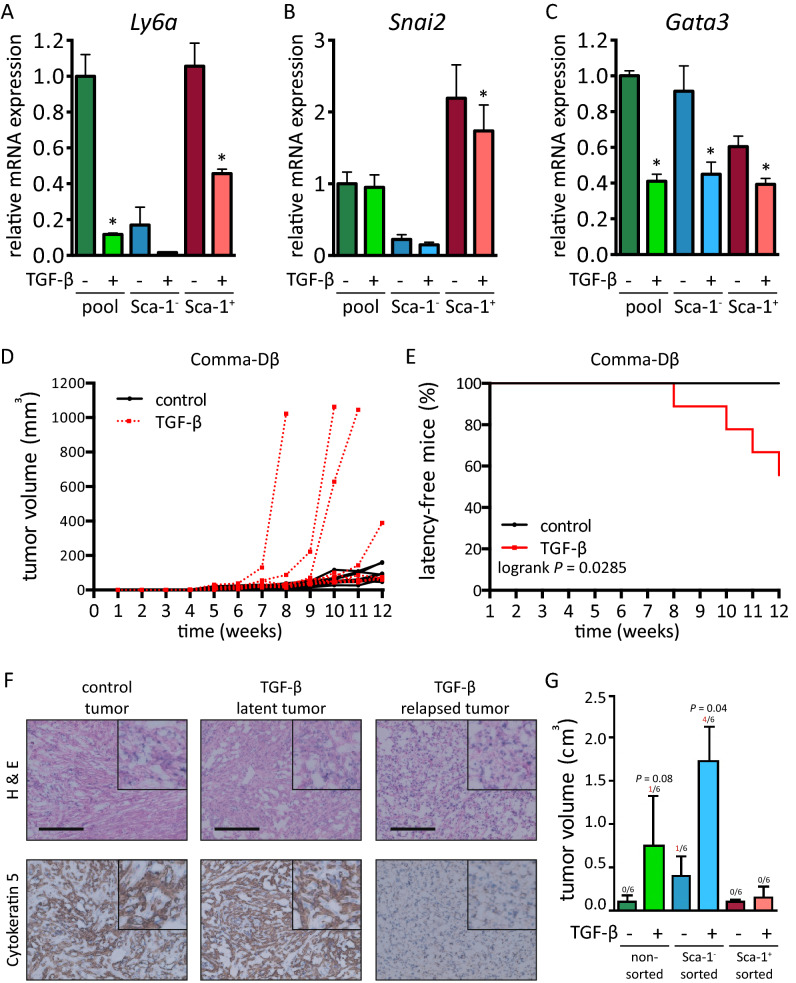
TGF-β induces de-differentiation and oncogenic transformation of mammary epithelial cells. **(A-C)** The plot shows changes in gene expression of Sca-1 mRNA (*Ly6a*), regulator of basal phenotype *Snai2*, and regulator of luminal phenotype *Gata3* in sorted subpopulations of Comma-Dβ cells, exposed to exposed to vehicle (PBS) or TGF-β1 (1 ng/mL) for 96 h directly after sorting. Results are presented as mean ± SD (paired *t* test or ratio-paired *t* test, *P < 0.05). **(D)** The plot shows single mouse tracks for tumor growth of Comma-Dβ cells, measured with calibrated digital caliper. Results are from two independent experiments, n = 9 mice *per* group. Cells were pretreated prior to implantation with vehicle or 1 ng/mL TGF-β1 for 72 h. **(E)** The plot shows % of mice without relapsing tumors and is related to panel (**D**; Mantel-Cox test). **(F)** Representative images showing tumor morphology (H&E) and Cytokeratin 5 staining in control and TGF-β1-pre-treated tumors. Refer to Supplementary Fig. S6D for a full panel of stainings and additional controls (scale = 200 μm). **(G)** Plot shows quantification of tumorigenic potential of non-sorted Comma-Dβ cells exposed to vehicle or TGF-β1 (1 ng/mL) for 72 h prior to implantation, and of Sca-1^−^ and Sca-1^+^ Comma-Dβ cells sorted from vehicle- or TGF-β1-treated Comma-Dβ cell line. Number or relapsing tumors is shown above corresponding error bars. Data were collected at week 8 post-implantation in two independent experiments and are visualized as mean ± SEM (n = 6 *per* group, Mann–Whitney *u* test).

The TGF-β1-induced perturbations in lineage-specific genes suggested that the TGF-β signaling affects the differentiation state of Comma-Dβ cells. The unexpected, concomitant decrease of master basal- (*Snai2* and *Sox9*) and luminal-lineage regulators (*Gata3*, see Fig. 2D) led us to further dissect the phenotypic changes in both cell lineages. We sorted the Sca-1^−/+^ fractions and exposed them to TGF-β1 (Fig. S6A-C). As expected, the Sca-1 and Slug mRNAs were down-regulated in sorted basal-like Sca-1^+^ fractions, while the Gata3 mRNA was decreased in sorted luminal-like Sca-1^−^ fraction (Fig. 4A–C).

In order to determine the contribution of short-term TGF-β1 exposure to the tumorigenicity of these pre-neoplastic cells, we exposed them to TGF-β1 prior to implantation into the mammary fat pad. Most of the tumors showed 4–6 weeks latency and grew rather slowly. However, a portion of tumors derived from TGF-β1 pre-treated cells (4/9) escaped this latency and formed relapsing tumors (Fig. 4D–E). These relapsed tumors were typical by high cell density, weak Cytokeratin 5 immunoreactivity^27^ and low content of collagen-containing extracellular matrix (Fig. 4F and S6D). Further sorting and in vivo implantation of TGF-β1 pre-treated cells showed, that the Sca-1- fraction of Comma-Dβ cells contained tumorigenic clones, and this was further enhanced with TGF-β1 (Fig. 4G). On the contrary, pre-treatment of bona fide cancer-stem like cell line ANV2 with TGF-β1 resulted in delayed tumor growth, reduced tumorigenicity (100% in vehicle vs. 87% in TGF-β1 pre-treated group) and prolonged overall survival (Fig. S5E-F). These observations confirm that TGF-β can be promoting tumor growth in pre-neoplastic cells while being tumor suppressive in cancer cells with functional TGF-β signaling. Since it was uncertain whether the endogenous mammary epithelium itself supports the sudden growth of relapsing tumors, we injected the Comma-Dβ cells into the cleared mammary gland. Both the vehicle and TGF-β1-pre-treated cells showed similar tumorigenic potential in the cleared mammary fat pad (Fig. S6G-H). However, the TGF-β1-pre-treated cells failed to form relapsing tumors in the lack of endogenous epithelium. The endogenous epithelium was thus necessary for this relapsing behavior of luminal-like Sca-1^−^ cells. The de-differentiation of pre-neoplastic mammary epithelial cells in response to TGF-β1 is accompanied by the enrichment or pre-selection of cells with increased tumor-initiating capacity.

### Sca-1 is the target gene of the TGF-β signaling pathway

**Figure 5:**
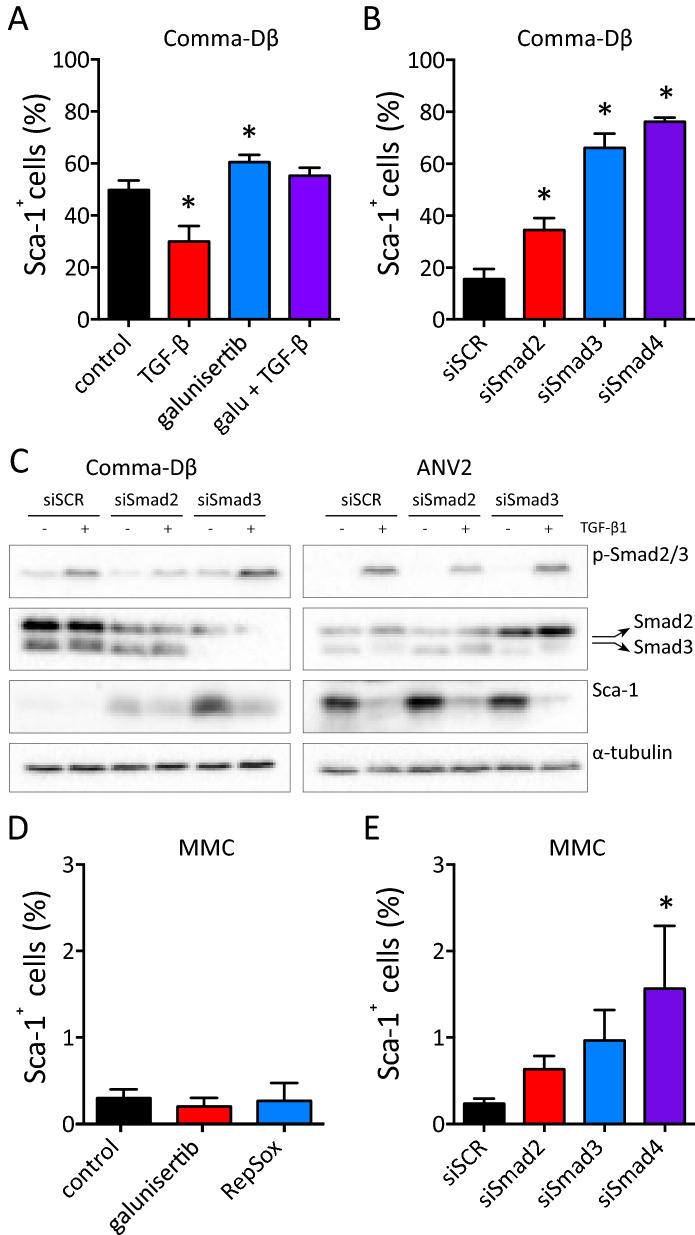
Sca-1 is a target gene of TGF-β pathway. **(A)** The plot shows % of cells positive for surface Sca-1 expression after experimental treatment. Comma-Dβ cells were pre-treated with vehicle (DMSO) or 5 μM galunisertib for 30 min and then with vehicle (PBS with 0.1% BSA) or 1 ng/mL TGF-β1 for further 72 h. The results are presented as mean ± SD from at least three independent experiments (*P < 0.05, *t* test) and were analyzed with flow cytometry. **(B)** The plot shows % of cells positive for surface Sca-1 expression after silencing of Smad2, Smad3 or Smad4 expression. Comma-Dβ cells were transfected with scrambled siRNA (100 nM) or siRNA against murine Smad2 (50 nM), Smad3 (50 nM) or Smad4 (100 nM) and collected after 72 h. The results are presented as mean ± SD from at least three independent experiments (*P < 0.05, *t* test) and were analyzed with flow cytometry. **(C)** The plot shows % of cells positive for surface Sca-1 expression after experimental treatment. MMC cells were exposed to vehicle (DMSO), 1 μM RepSox or 5 μM galunisetib for 72 h. The results are presented as mean ± SD from at least three independent experiments and were analyzed with flow cytometry. **(D)** The plot shows % of cells positive for surface Sca-1 expression after silencing of Smad2, Smad3 or Smad4 expression. MMC cells were transfected with scrambled siRNA (100 nM) or siRNA against murine Smad2 (50 nM), Smad3 (50 nM) or Smad4 (100 nM) and collected after 72 h. The results are presented as mean ± SD from at least three independent experiments and were analyzed with flow cytometry (*P < 0.05, *t* test).

We further evaluated the molecular basis of Sca-1 repression through TGF-β. Pre-treatment of cells with galunisertib, an ALK5/TGFBR1 inhibitor, before the addition of TGF-β1 rescued Sca-1 repression (Fig. 5A and S7A). This indicated an ALK5-dependent mechanism. Silencing of ALK5 downstream signal transducers Smad2, Smad3, and Smad4 resulted in an increased number of Sca-1^+^ cells (Fig. 5B and S7B). However, both Smad2 and Smad3 were dispensable for Sca-1 repression upon the exposure to TGF-β1 (Fig. 5C). Interestingly, while the inhibition of ALK5 did not stimulate the Sca-1 expression in Sca-1-negative MMC cells (Fig. 5D and S7C), the silencing of Smad4 generated a subpopulation of Sca-1^+^ cells (Fig. 5E).

**Figure 6:**
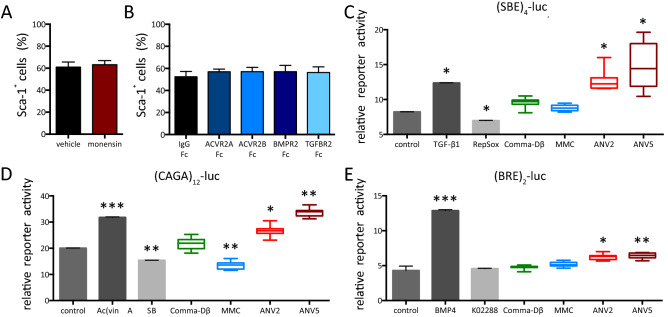
Sca-1 expression is not regulated in an autocrine manner in Comma-Dβ cells. **(A)** The plot shows % of cells positive for surface Sca-1 expression after experimental treatment. Comma-Dβ cells were exposed to vehicle (water) or 200 nM monensin for 72 h. The results are presented as mean ± SD from at least three independent experiments and were analyzed with flow cytometry. **(B)** The plot shows % of cells positive for surface Sca-1 expression after exposure of Comma-Dβ cells to vehicle (PBS with 0.1% BSA or IgG-Fc) or 1 μM of selected Type II receptor chimeras for 72 h. The results are presented as mean ± SD from two independent experiments and were analyzed with flow cytometry. **(C)** The plot shows relative reporter activity in HEK293 cells, co-transfected with (SBE)_4_-luc and *Renilla* vector, starved for 4 h in serum-free medium and exposed to control treatment (DMSO, 1 ng/mL TGF-β1 or 1 μM RepSox) or conditioned media (Comma-Dβ, MMC, ANV2 or ANV5) for 2 h. Data are from two (controls) or four technical replicates (conditioned media). Three biological replicates of conditioned media *per* cell line were analyzed (*P < 0.05, *t* test). **(D)** The plot shows relative reporter activity in HEK293 cells, co-transfected with (CAGA)_12_-luc and *Renilla* vector, starved for 4 h in serum-free medium and exposed to control treatment (DMSO, 1 ng/mL Activin A or 1 μM SB431542) or conditioned media (Comma-Dβ, MMC, ANV2 or ANV5) for 2 h. Data are from two (controls) or four technical replicates (conditioned media). Three biological replicates of conditioned media *per* cell line were analyzed (*P < 0.05, *t* test). **(E)** The plot shows relative reporter activity in HEK293 cells, co-transfected with (BRE)_2_-luc and *Renilla* vector, starved for 4 h in serum-free medium and exposed to control treatment (DMSO, 10 ng/mL BMP4 or 1 μM K02288) or conditioned media (Comma-Dβ, MMC, ANV2 or ANV5) for 2 h. Data are from two (controls) or four technical replicates (conditioned media). Three biological replicates of conditioned media per cell line were analyzed (*P < 0.05, *t* test).

TGF-β ligands are important components of cell secretome that can potentially affect the lineage commitment in an autocrine or paracrine manner. We focused on their role in the maintenance of basal-to-luminal (Sca-1^+^-to-Sca-1^−^) equilibrium in the Comma-Dβ cell line. The inhibition of protein transport from the Golgi apparatus outside of the cell with a sublethal dose of monensin did not disrupt this equilibrium (Fig. 6A). We further specifically neutralized several ligands-of-interest using Type II receptor Fc-chimeras (Fig. 6B and S8A-D). Such inhibition of TGF-β ligands did not affect the ratio of basal- and luminal-like cells and the Sca-1 levels (Fig. S7B), except for the up-regulation of Sca-1 in ANV5 cells, that produce high levels of active TGF-β1 (Fig. S8E), upon exposure to TGFBR2-Fc. The conditioned media from ANVs also showed high activation of Smad2/3 (TGF-β1 and Activin) reporters and moderate activation of BMP reporter (Fig. 6C–E). These results confirmed that the components of the TGF-β signaling pathway modulate the expression of Sca-1 in a cell type-specific manner: while the basal-to-luminal ratio in Comma-Dβ was not affected by its own TGF-β family ligands, the TGF-β1 secreted by ANV cancer stem cells changed the expression of its target gene Sca-1 in the autocrine fashion.

## Discussion

TGF-β signaling plays a critical role in the development and homeostasis maintenance of mammary gland^28^. To assess the effect of TGF-β on Sca-1 expression in pathophysiology, we used the pre-neoplastic mammary epithelial cells Comma-Dβ. This cell line is known for its heterogeneity: the presence of the basal-like subpopulation that exhibits progenitor capability and expresses Sca-1, beside the luminal-like cells that are negative for Sca-1 expression^23^. We showed that the exposure of these cells to TGF-β induced the loss of Sca-1 expression and drove the de-differentiation of both cell lineages. In the normal mammary gland, TGF-β signals are exacerbated during involution and induce apoptosis of unnecessary epithelial cells in branched mammary gland^29^. Comma-Dβ cell line contains clones with mutant Tp53^27,30^, likely responsible for its immortality and pre-neoplastic properties of Sca-1^−^ subpopulation. Implantation of these cells, pre-treated with TGF-β, into mammary fat pad resulted in shorter latency of tumor growth and sudden relapses. This phenomenon was previously shown only for cells that were exposed to TGF-β for relatively long periods of time^31^. However, our results confirmed that even the transient, short-term exposure to low TGF-β concentrations sufficiently induces the accumulation of tumor-initiating cells through an unknown mechanism. A similar experiment using mesenchymal cancer-stem cells with functional TGF-β pathway showed that TGF-β has as well tumor-suppressive action. Altogether, we provide evidence that TGF-β can promote or suppress tumorigenesis in a context-dependent manner.

Mechanistically, we proposed that Sca-1 was repressed by the endogenous TGF-β signaling and was re-expressed after the inhibition of ALK5, the Type I TGF-β receptor, or by the knock-down of its downstream signal transducers Smad2/3/4. This effect was independent of the ALK5 kinase activity in luminal-like, Her2-overexpressing cancer cells and only the silencing of Smad4 led to the generation of Sca-1 positive cells. In contrary to myogenic cells, Smad2 and Smad3 were dispensable for Sca-1 repression in mammary epithelial and cancer cells, mediated by exogenous TGF-β, possibly specific for the mammary gland^32^. These results, thus, confirm that the regulation of Sca-1 expression on the cell surface of mammary epithelial and cancer cells is cell lineage-specific and governed by the components of the TGF-β family signaling.

## Supplementary information

Supplementary file1

Supplementary file2
