# Supplementary material for: TGF-β regulates Sca-1 expression and plasticity of pre-neoplastic mammary epithelial stem cells
Source: Sci Rep. 2020 Jul 9;10:11396. doi: 10.1038/s41598-020-67827-4 (PMC7347574; doi:10.1038/s41598-020-67827-4)
Supplement: Supplementary file 1 — Supplementary file1 [file 41598_2020_67827_MOESM1_ESM.pdf]

TGF-β regulates Sca-1 expression and plasticity of pre-neoplastic mammary epithelial stem cells

Ján Remšík, Markéta Pícková, Ondrej Vacek, Radek Fedr, Lucia Binó, Aleš Hampl, and Karel Souček

Supplementary Figures

Supplementary Figure 1

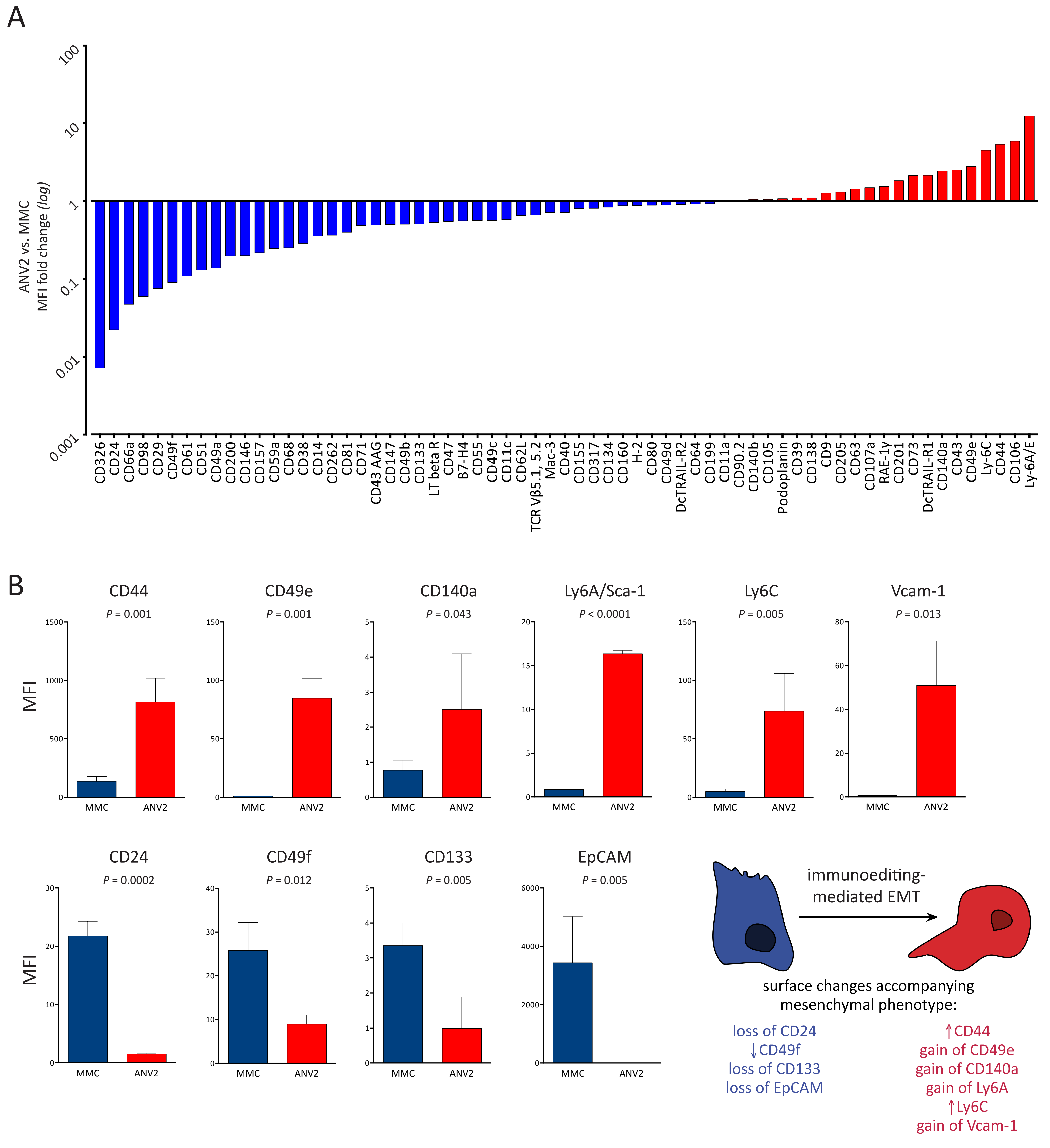

Supplementary Figure 1. Surface profile of MMC and ANV2 cells.

(A) The plot shows expression pattern of surface markers in MMC and ANV2 cells, acquired with high-throughput surface profiling. Data are shown as *log* fold change (MFI = median fluorescence index).
(B) The plot shows independent validations of selected, differentially expressed surface markers in MMC and ANV2 cells, identified with high-throughput surface profiling and analyzed with flow cytometry. Results are from at least three independent experiments and are presented as mean ± SD, paired *t* test (MFI = median fluorescence index).

Supplementary Figure 2

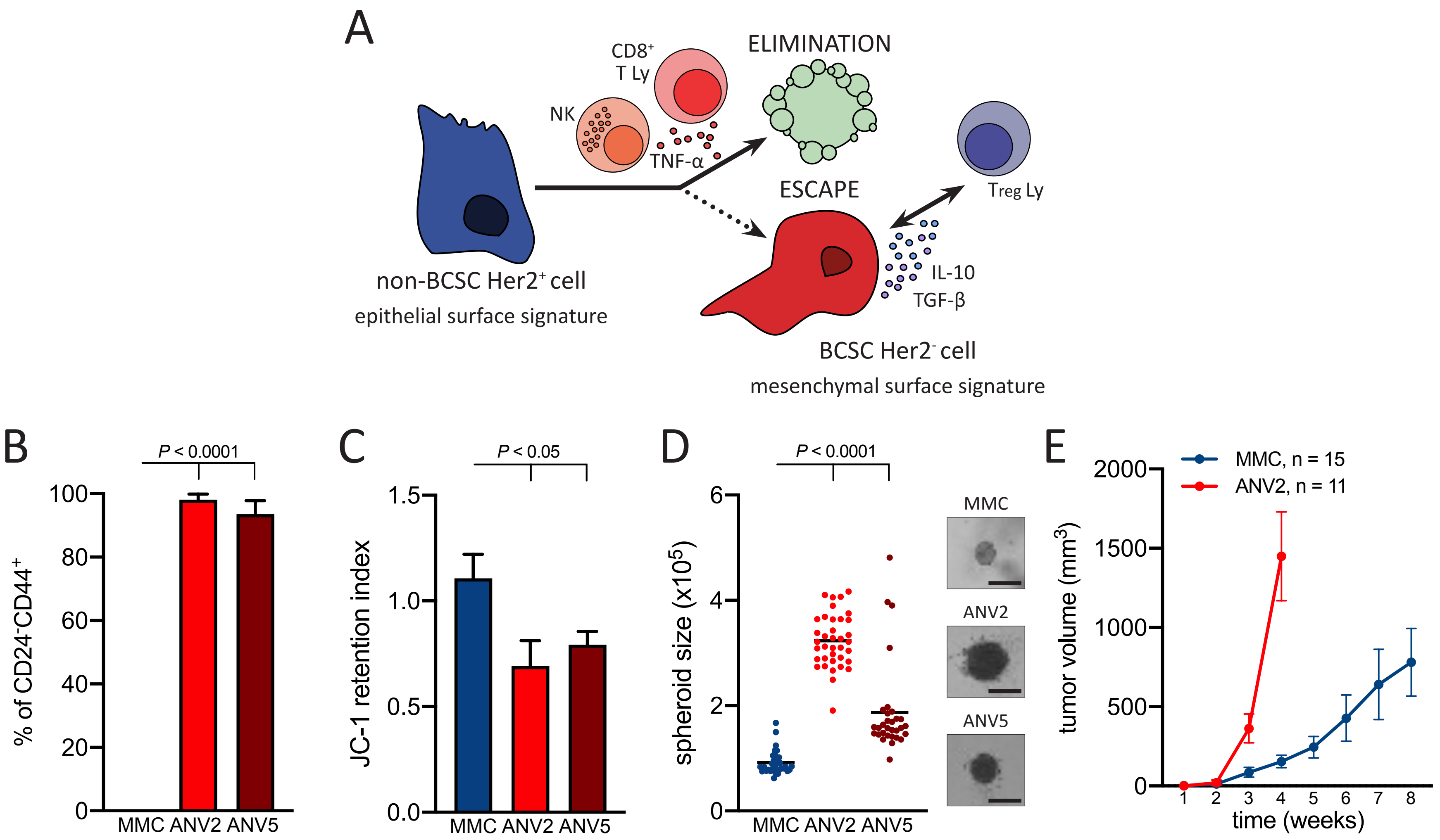

Supplementary Figure 2. Stem cell characteristics of ANV2 cells.

(A) The scheme shows an establishment of experimental models MMC and ANV2, as described in Knutson *et al*., *The JI* 2006;117(3).
(B) Plot shows the percentage of breast cancer stem-like cells, as characterized by CD24-CD44+ immunophenotype, in the culture of MMC, ANV2 and ANV5 cells. Three biological replicates *per* cell line were analysed with flow cytometry and are shown as mean ± SD (*t* test).
(C) Plot shows capacity of MMC, ANV2 and ANV5 cells to retain JC-1 as a proxy of ABC transporter activity in standard cell culture conditions. Results are from four measurements *per* subline and are presented as mean ± SD (*t* test).
(E) Representative images and scatter plot show spheroid size of MMC, ANV2 and ANV5 cell lines in standard cell culture conditions, as determined with spheroid formation assay. Results are from two independent experiments (Mann-Whitney *u* test, scale = 500 µm).
(F) Plot shows *in vivo* behaviour of MMC (n = 15; $2.5x10^5$ cells injected *per* mouse) and ANV2 cells (n = 14; $5x10^4$ cells injected *per* mouse) implanted into mammary fat pad. The growth curves were derived from plots shown in Figure 2F (MMC, EV clones) and Supplementary Figure 6E (ANV2, vehicle). Results are presented as mean ± SEM.

Supplementary Figure 3

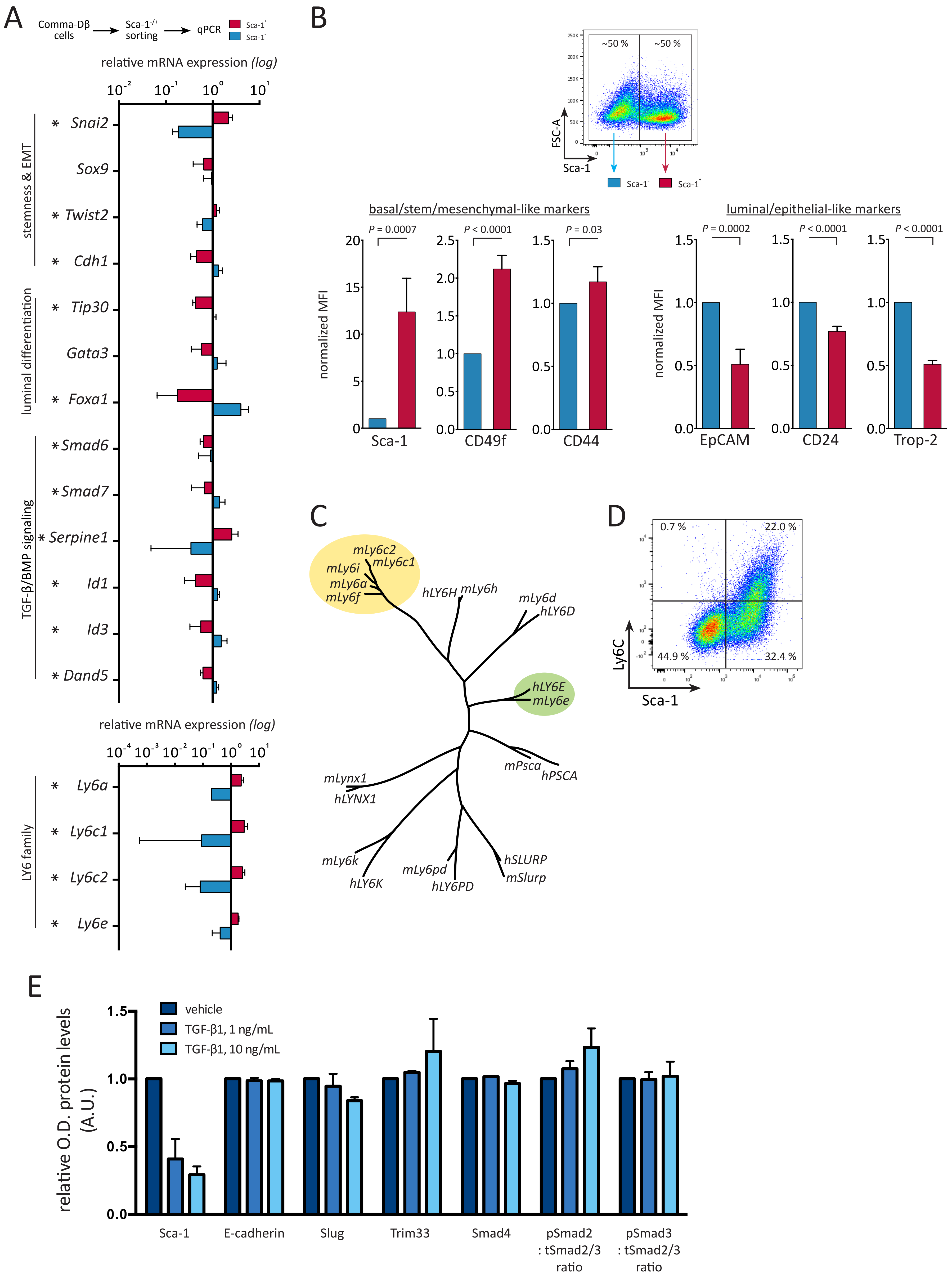

Supplementary Figure 3. Characterization of Comma-Dβ Sca-1$^{-/+}$ subpopulations.

(A) Plot shows gene expression pattern of stemness & EMT associated genes, luminal phenotype regulators and selected genes from TGF-β/BMP pathway in sorted Sca-1$^{-/+}$ fractions, sorted from Comma-Dβ cells. Results are from four independent experiments and are presented as mean ± SD in *log* scale (multiple *t* tests, * FDR $q < 0.05$).
(B) Plots show relative abundance of selected surface antigens on the cell membrane of Sca-1$^{-/+}$ Comma-Dβ fractions. Results are from at least three independent experiments, analyzed with flow cytometry and are presented as mean ± SD (MFI = median fluorescence index).
(C) Phylogenetic tree shows relation of protein sequences for murine LY6 family members and their known human orthologs. The cluster of closer relatives, analyzed in panel (A), is highlighted in yellow, while the more distant relative Ly6e is highlighted in green.
(D) Representative dot plot from three independent experiments shows co-expression of Sca-1 and Ly6C in Comma-Dβ cells. Data are related to panel (B).
(E) Densitometric quantification of western blot shown in Figure 3C.

Supplementary Figure 4

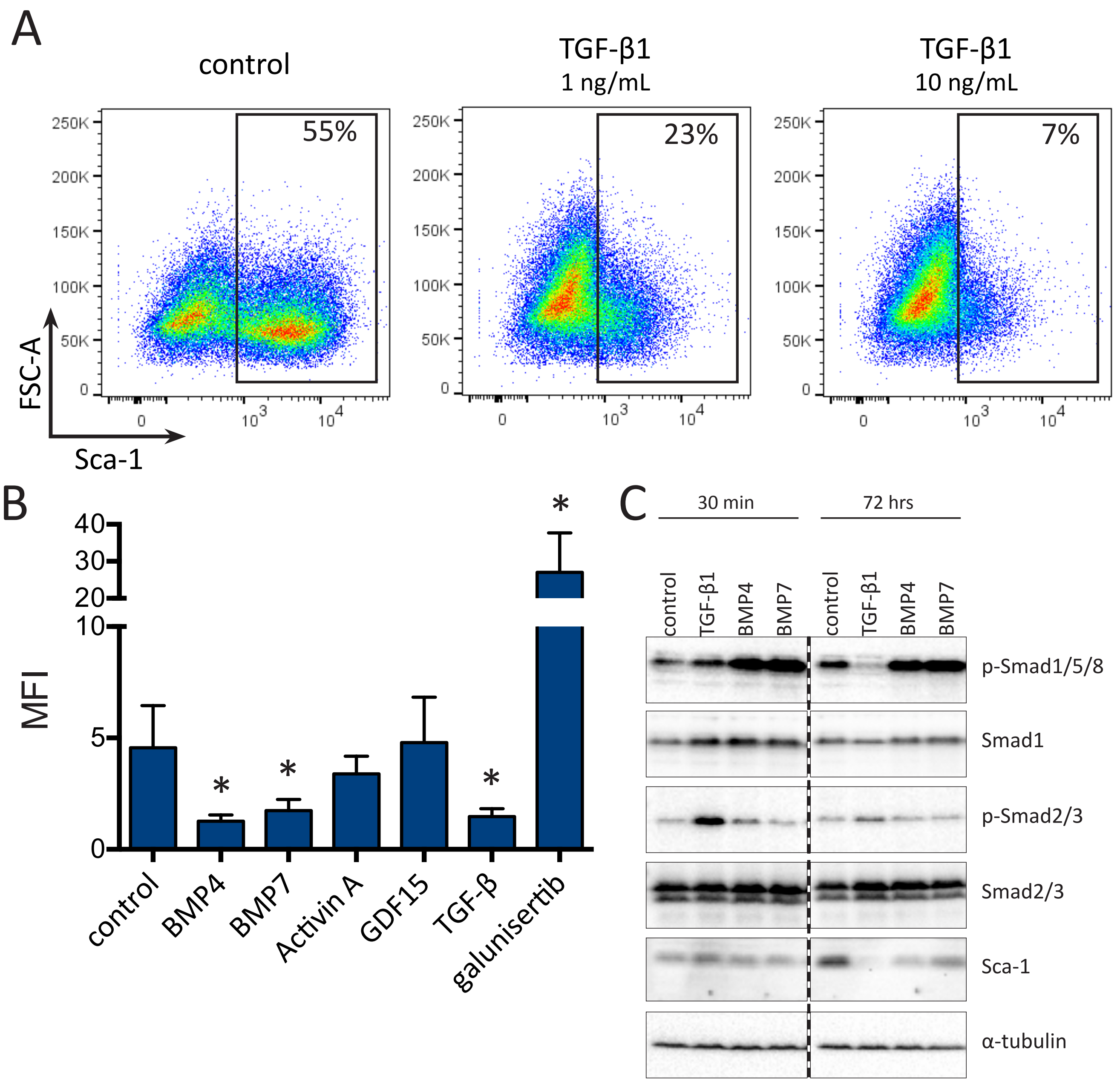

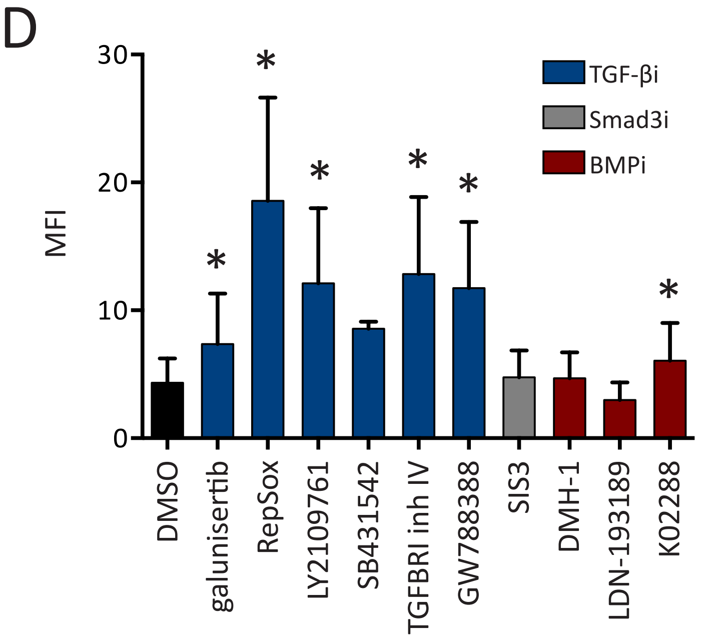

Small molecule inhibitor	Target	Pathway
Galunisertib (1 µM)	ALK5	TGF-β
RepSox (1 µM)	ALK5	TGF-β
LY2109761 (1 µM)	ALK5, TGFBR2	TGF-β
SB431542 (1 µM)	ALK4, ALK5, ALK7	TGF-β, Nodal, Activin
TGFBRI kinase inhibitor IV (1 µM)	ALK4, ALK5, ALK7	TGF-β, Nodal, Activin
GW788388 (1 µM)	ALK5, TGFBR2, ACVR2	TGF-β, Nodal, Activin (BMP)
SIS3 (1 µM)	SMAD3	TGF-β
DMH-1 (1 µM)	ALK2	BMP
LDN-193189 (0.25 µM*)	ALK2, ALK3	BMP
K02288 (1 µM)	ALK1, ALK2, ALK3	BMP
Dorsomorphin (1 µM)	AMPK, ALK2, ALK3, ALK6	BMP

*lethal at 1 µM

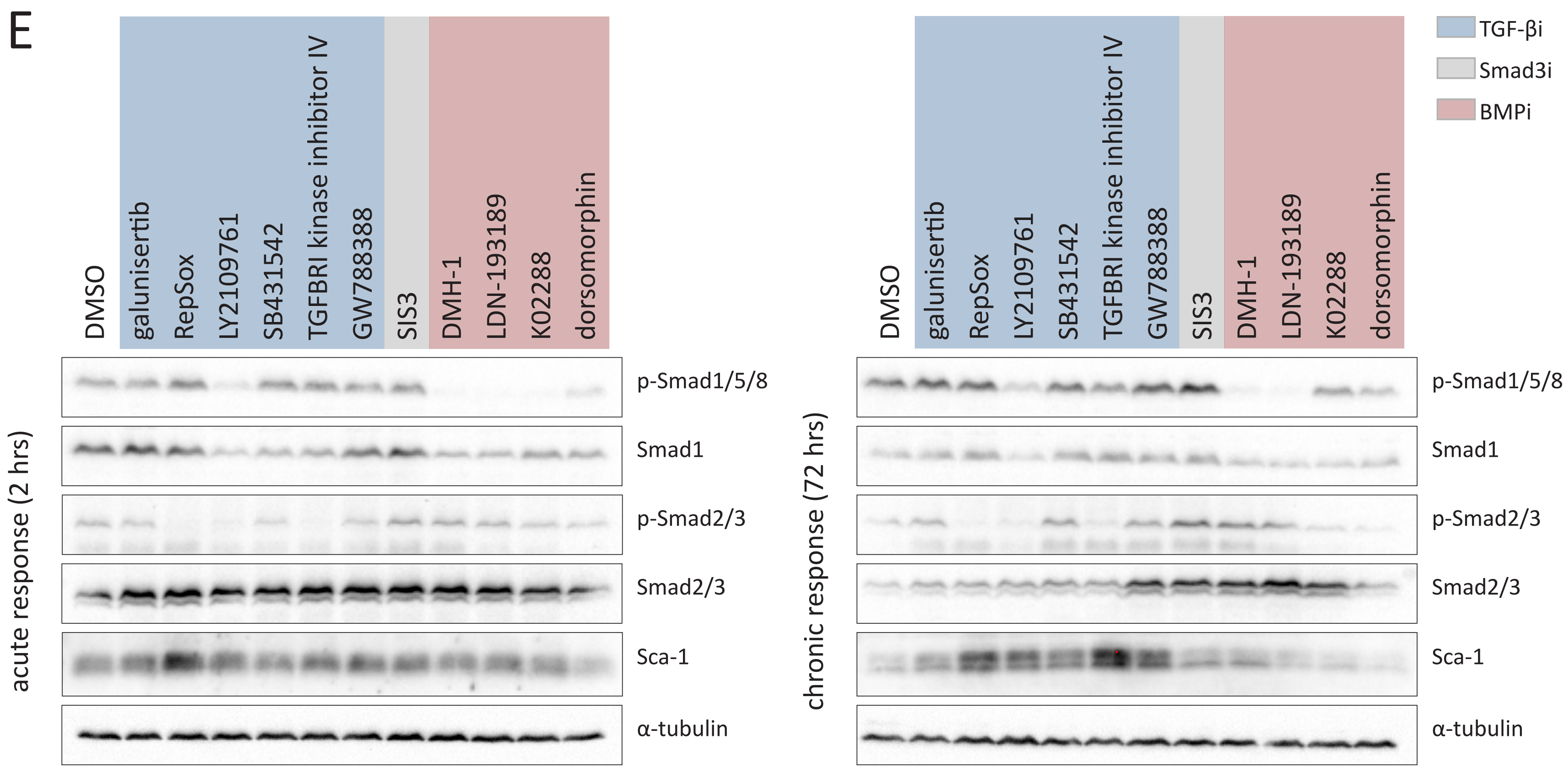

Supplementary Figure 4. Regulation of Sca-1 expression through TGF-β.

(A) Representative dot plots from at least three independent experiments show decrease of Sca-1^+ Comma-Dβ cells upon 72 h exposure to selected concentrations of TGF-β1.

(B) Plot shows down-regulation of surface Sca-1 expression upon exposure of Comma-Dβ cells to vehicle (PBS with 0.1% BSA), BMP4 (100 ng/mL), BMP7 (100 ng/mL), Activin A (10 ng/mL), GDF15 (200 ng/mL), TGF-β1 (1 ng/mL) and galunisertib (5 µM) for 72 h. Data are from three independent experiments, analyzed with flow cytometry and presented as mean ± SD, (*$P < 0.05$, *t* test; MFI = median fluorescence index).

(C) Representative western blots show activation of TGF-β and BMP pathways upon short-term (30 min) or long-term (72 h) exposure of Comma-Dβ cells to vehicle (PBS), TGF-β1 (1 ng/mL), BMP4 (100 ng/mL) and BMP7 (100 ng/mL), in relation to panel (B).

(D) Plot shows changes in surface Sca-1 expression upon exposure of Comma-Dβ cells to vehicle (DMSO) or selected inhibitors of TGF-β and BMP pathways, summarized in the table. Data are from three independent experiments, analyzed with flow cytometry and presented as mean ± SD (*$P < 0.05$, *t* test; MFI = median fluorescence index).

(E) Representative western blots show inhibition of Smad phosphorylation upon acute (2 h) or chronic (72 h) exposure of Comma-Dβ cells to vehicle (DMSO) or selected inhibitors of TGF-β and BMP pathways, in relation to panel (D).

Supplementary Figure 5

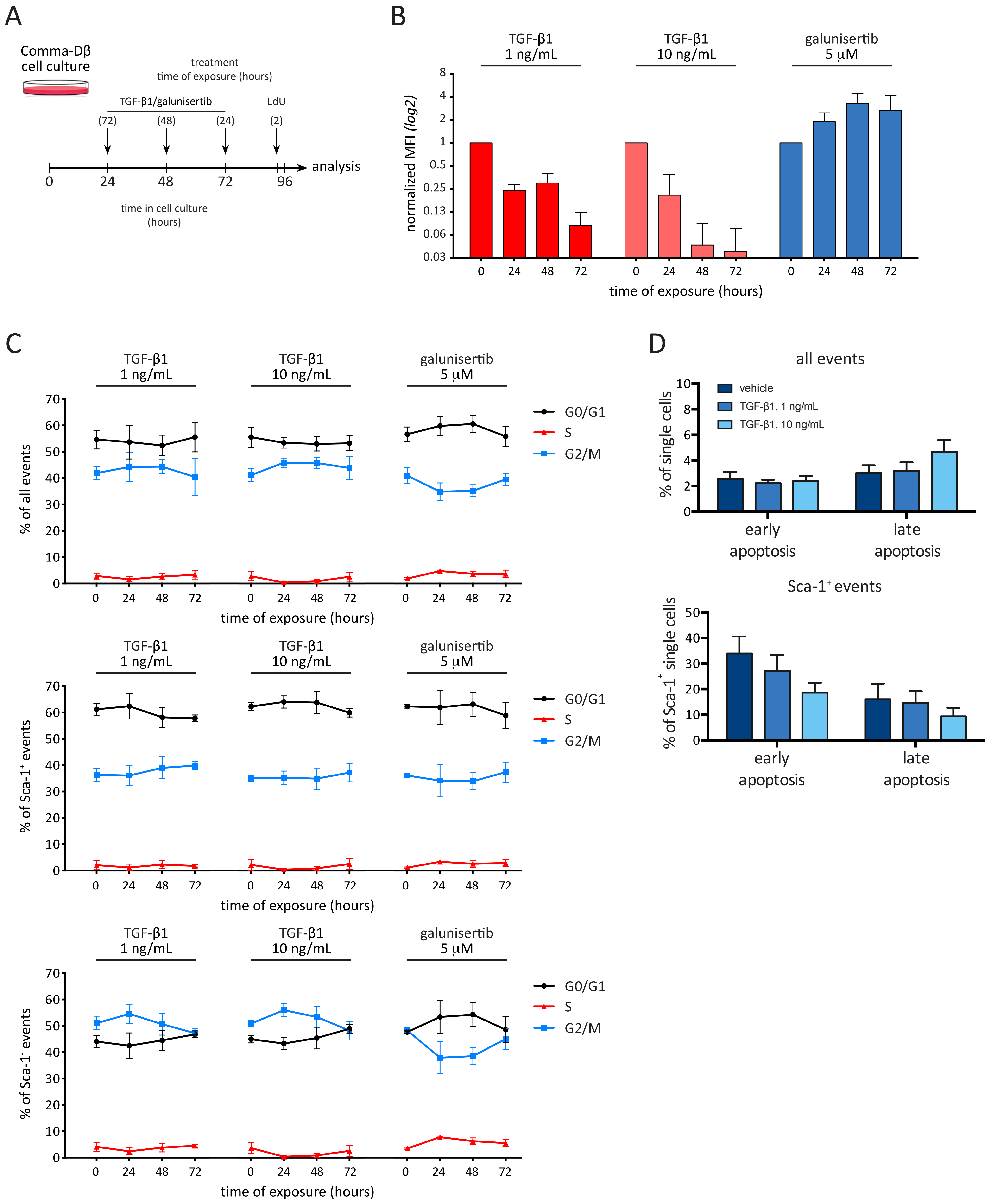

Supplementary Figure 5. Short-term TGF-β1 exposure does not induce apoptosis and cell cycle changes in Comma-Dβ cells.

(A) Scheme shows experimental workflow of kinetic experiment, used for determination of changes in cell cycle and Sca-1 expression in Comma-Dβ cells.
(B) Plot shows relative changes in Sca-1 expression upon exposure to experimental treatment. Data are from three independent experiments, analyzed with flow cytometry and presented as mean ± SD (MFI = median fluorescence index).
(C) Plots show changes in cell cycle of whole cell population (upper plot), Sca-1^{+} cells only (middle plot) or Sca-1^{-} cells only (lower plot) of Comma-Dβ cells upon exposure to experimental treatment. Data are from three independent experiments, analyzed with flow cytometry and presented as mean % of cells in denoted cell cycle phase ± SD. Cell cycle phases were resolved using DNA stain and proliferation marker EdU.
(D) Plots show changes in early (ANX^{+}/PI^{-}) and late apoptosis (ANX^{+}/PI^{+}) of whole cell population (upper plot), Sca-1^{+} cells only (bottom plot) of Comma-Dβ cells upon exposure to experimental treatment. Data are from three independent experiments, analyzed with flow cytometry and presented as mean % of cells ± SD.

Supplementary Figure 6

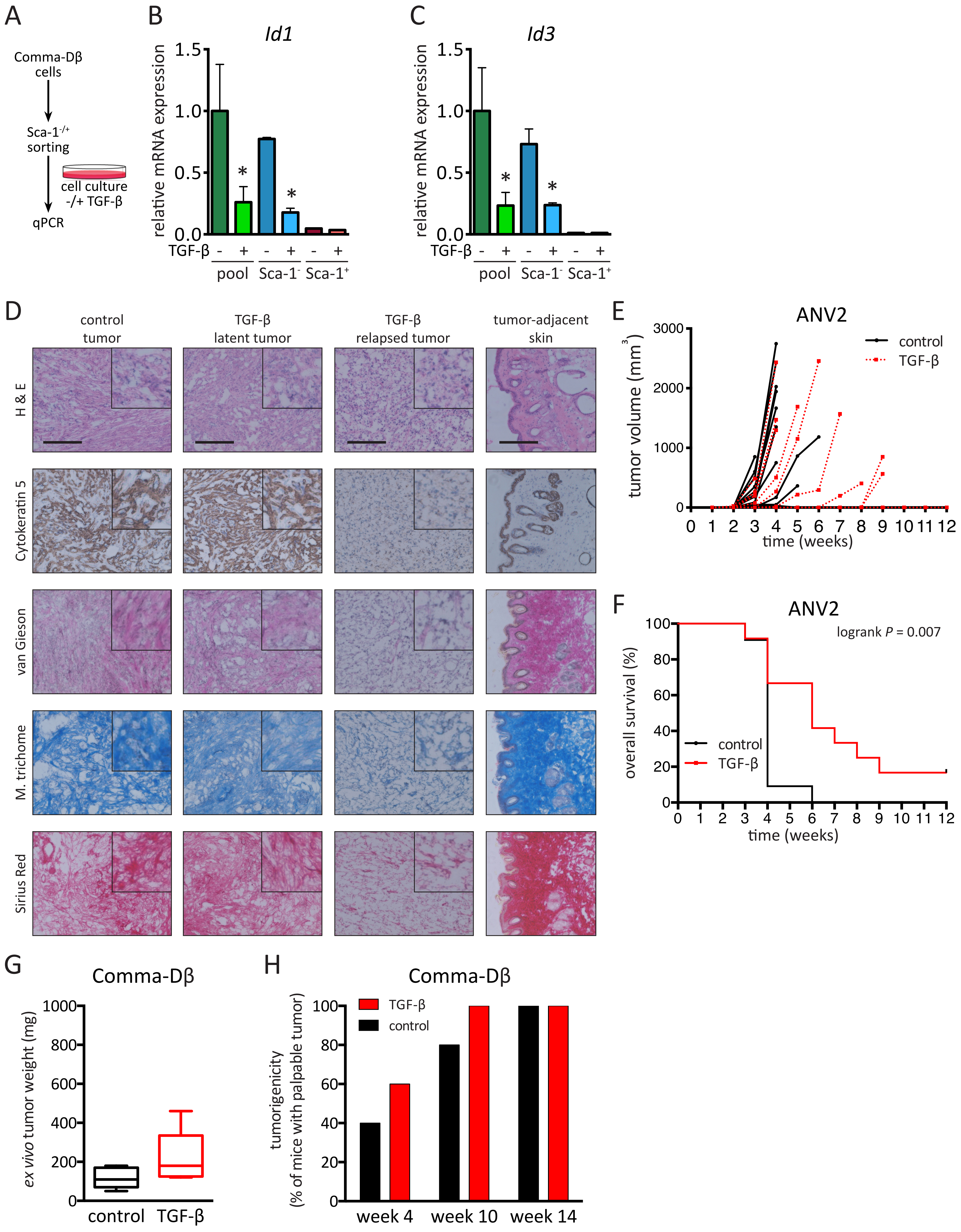

Supplementary Figure 6. Subpopulation-specific TGF-β1-induced changes in Comma-Dβ.

(A) Scheme shows experimental workflow, used for determination of gene expression changes upon exposure to experimental treatment for 96 h. Sca-$1^{+/-}$ fractions were sorted, washed and seeded into cell culture media containing vehicle (PBS) or TGF-β1 (1 ng/mL). Viable, single cells (pool) were sorted as controls and processed in the same way.
(B-C) Plot shows changes in gene expression of TGF-β target genes *Id1* and *Id3* in sorted subpopulations of Comma-Dβ cells, exposed to vehicle (PBS) or TGF-β1 (1 ng/mL) for 96 h directly after sorting. Results are presented as mean ± SD (paired *t* test, $*P < 0.05$).
(D) Representative images showing tumor morphology (H&E), expression of Cytokeratin 5 and collagen fibers (van Gieson, Massone's trichome and Sirius Red stains) in Comma-Dβ tumors formed by control and TGF-β-pretreated cells (scale = 200 μm).
(E) Plot shows *ex vivo* tumor weight, established in cleared fat pads of immunodeficient females 14 weeks after implantation of vehicle (PBS) or TGF-β1 (1 ng/mL) pre-treated (72 h) Comma-Dβ cells, n = 5 mice *per* group.
(F) Plot shows percentage of mice with palpable tumor in cleared fat pad; 4, 10 and 14 weeks after implantation. Data are related to panel (D).
(G) Plot shows single mouse tracks for tumor growth of ANV2 cells, measured with calibrated digital caliper. Results are from two independent experiments, n = 14-15 mice *per* group. Cells were pretreated prior to implantation with vehicle or 1 ng/mL TGF-β1 for 72 h.
(H) Plot shows percent of overall survival of mice in panel Fig. S5G (D; Mantel-Cox test).

Supplementary Figure 7

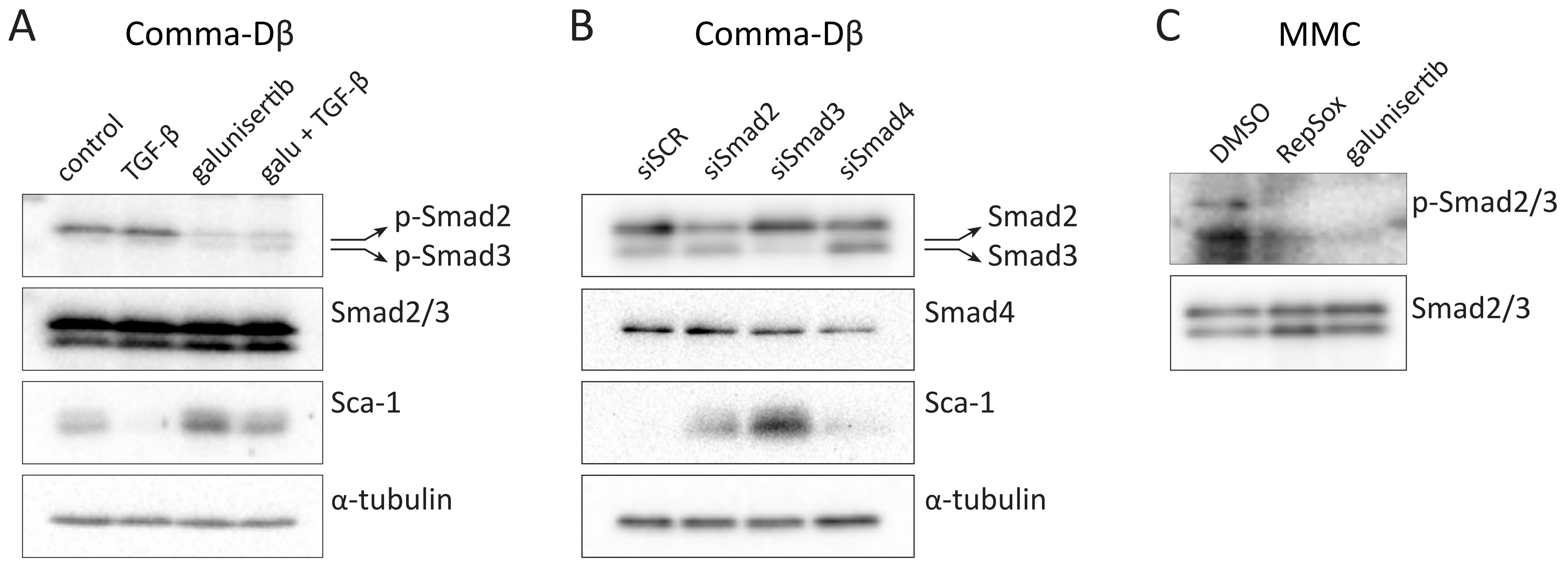

Supplementary Figure 7. Mechanism of Sca-1 expression regulation through TGF-β.

(A) Representative western blots show changes in Sca-1 levels upon exposure of Comma-Dβ cells to TGF-β1, galunisertib or their combination. Cells were pre-treated with vehicle (DMSO) or 5 μM galunisetib for 30 min and then with vehicle (PBS with 0.1% BSA) or 1 ng/mL TGF-β1 for further 72 h. Data are from three independent experiments. Blots show levels of phospho-Smad2/3, total Smad2/3, Sca-1 and α-tubulin. Data are from three independent experiments.

(B) Representative western blots show changes in Sca-1 expression after silencing of Smad2, Smad3 or Smad4. Comma-Dβ cells were transfected with scrambled siRNA (100 nM) or siRNA against murine Smad2 (50 nM), Smad3 (50 nM) or Smad4 (100 nM) and collected after 72 h. Data are from three independent experiments. Blots show levels of Smad2/3, Smad4, Sca-1 and α-tubulin. Data are from three independent experiments.

(C-D) Representative western blots show changes in Sca-1 levels after transfection of Comma-Dβ (C) or ANV2 (D) cells with scrambled or Smad2- or Smad3-specific siRNAs (all 50 nM). 4 h after transfection, cells were treated with vehicle (PBS with 0.1% BSA) or TGF-β1 (10 ng/mL) and collected 48 h after. Blots show levels of phospho-Smad2/3, total Smad2/3, Sca-1 and α-tubulin. Data are from three independent experiments.

(E) Representative western blot shows inhibition of Smad2/3 phosphorylation upon exposure of MMC cells to vehicle (DMSO), RepSox (1 μM) and galunisertib (5 μM) for 72 h. Blots show levels of phospho-Smad2/3 and total Smad2/3. Data are from two independent experiments.

Supplementary Figure 8

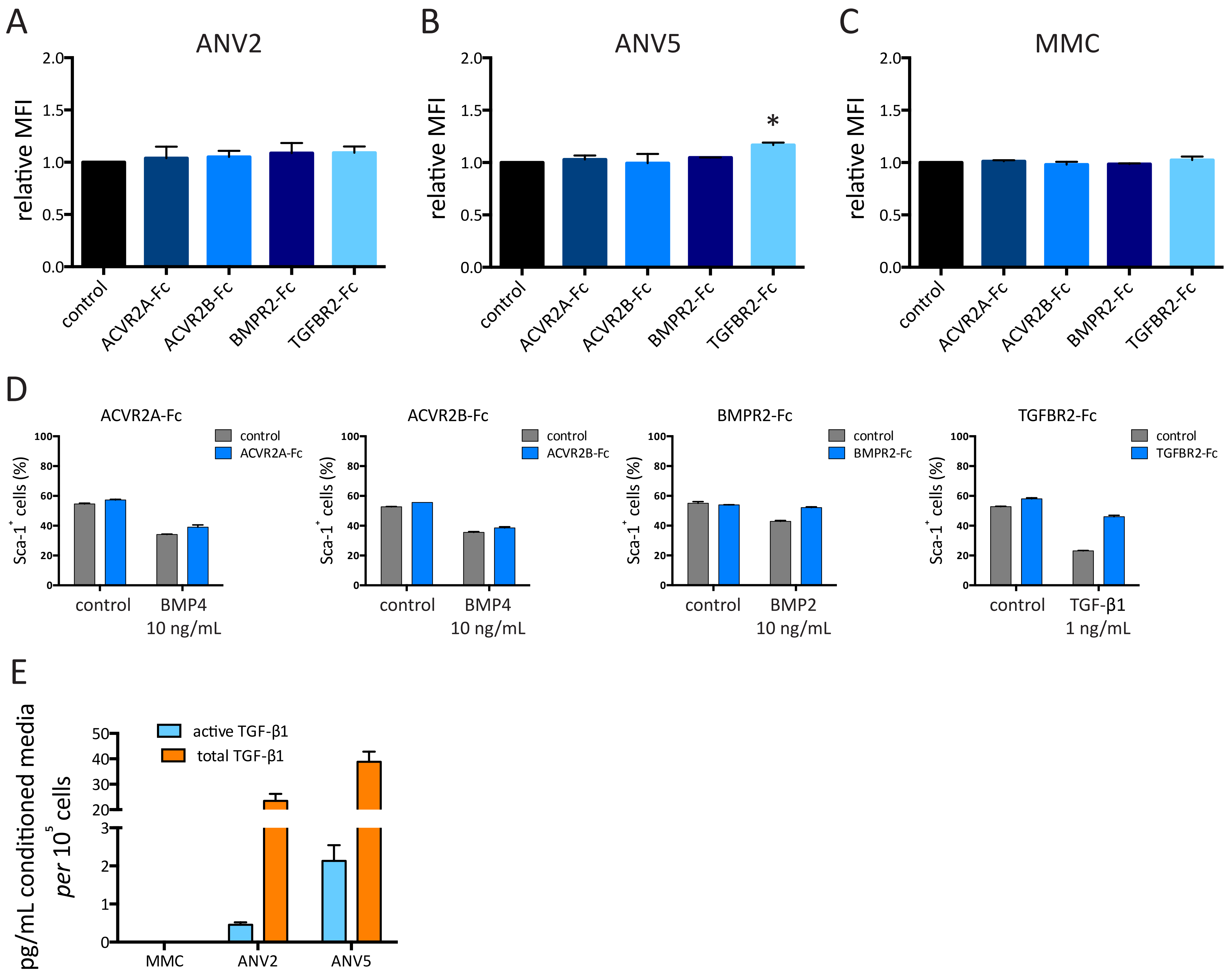

Supplementary Figure 8. Autocrine and paracrine regulation of Sca-1 expression.

(A-C) Plot shows relative surface Sca-1 expression after exposure of ANV2, ANV5 and MMC cells to vehicle (PBS with 0.1% BSA) or 1 μM of selected Type II receptor chimeras for 72 h. The results are presented as mean ± SD from two independent experiments (*$P < 0.05$) and were analyzed with flow cytometry (MFI = median fluorescence index).
(D) Plots show % of Sca-1 Comma-Dβ cells in validation of recombinant ligand quenching by receptor chimeras. Cells were exposed to vehicle (PBS with 0.1% BSA), ligands and/or chimeras for 72 h. The results are presented as mean ± SD from two technical replicates and were analyzed with flow cytometry.
(E) Plot shows concentration of active and total TGF-β1, as determined by ELISA in serum-free cell culture medium, condition by MMC, ANV2 and ANV5 cells for 24 h. Results are from four biological replicates.

Supplementary Figure 9

Uncropped membrane scans related to Fig. 2:

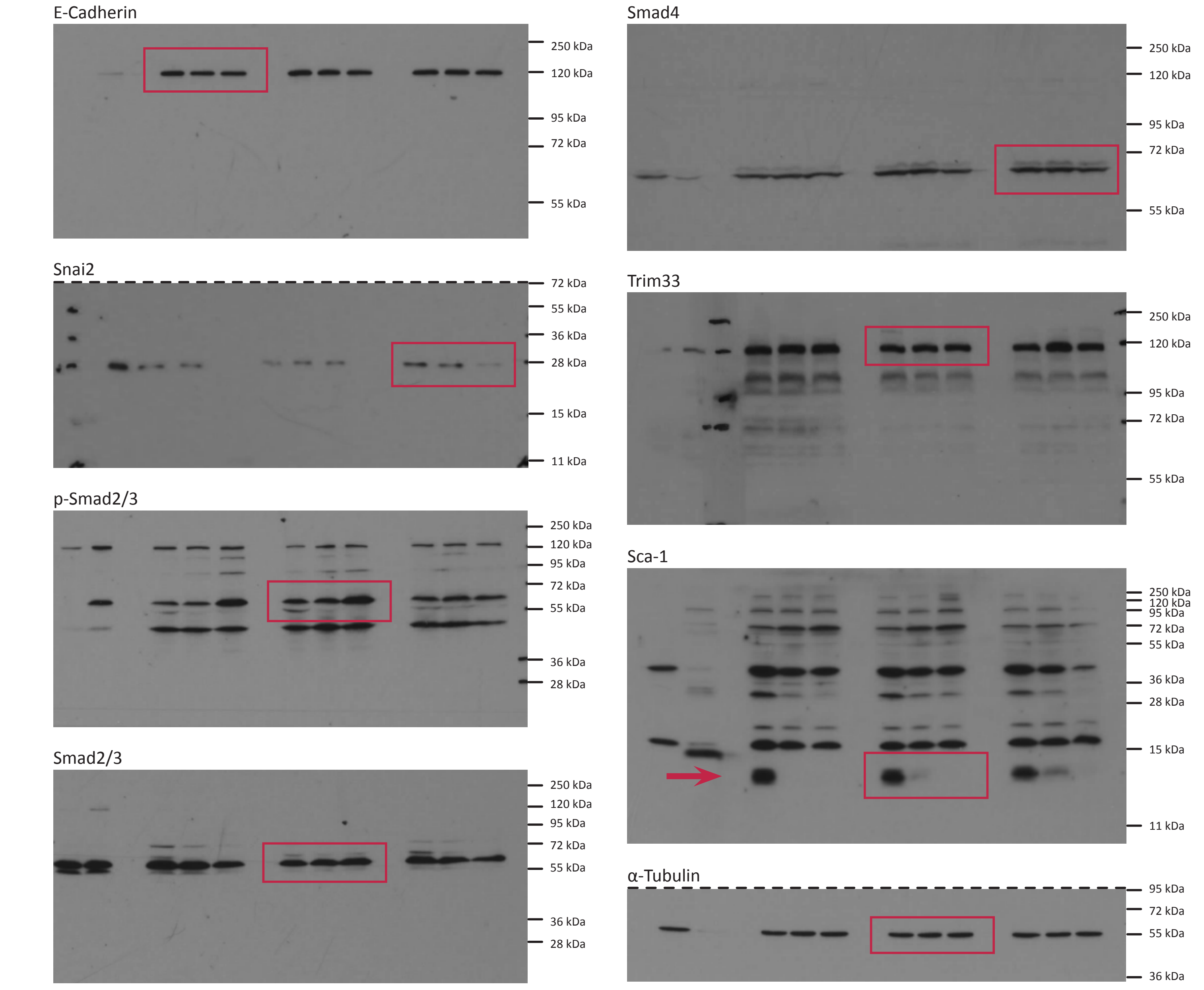

Uncropped membrane scans related to Fig. S3:

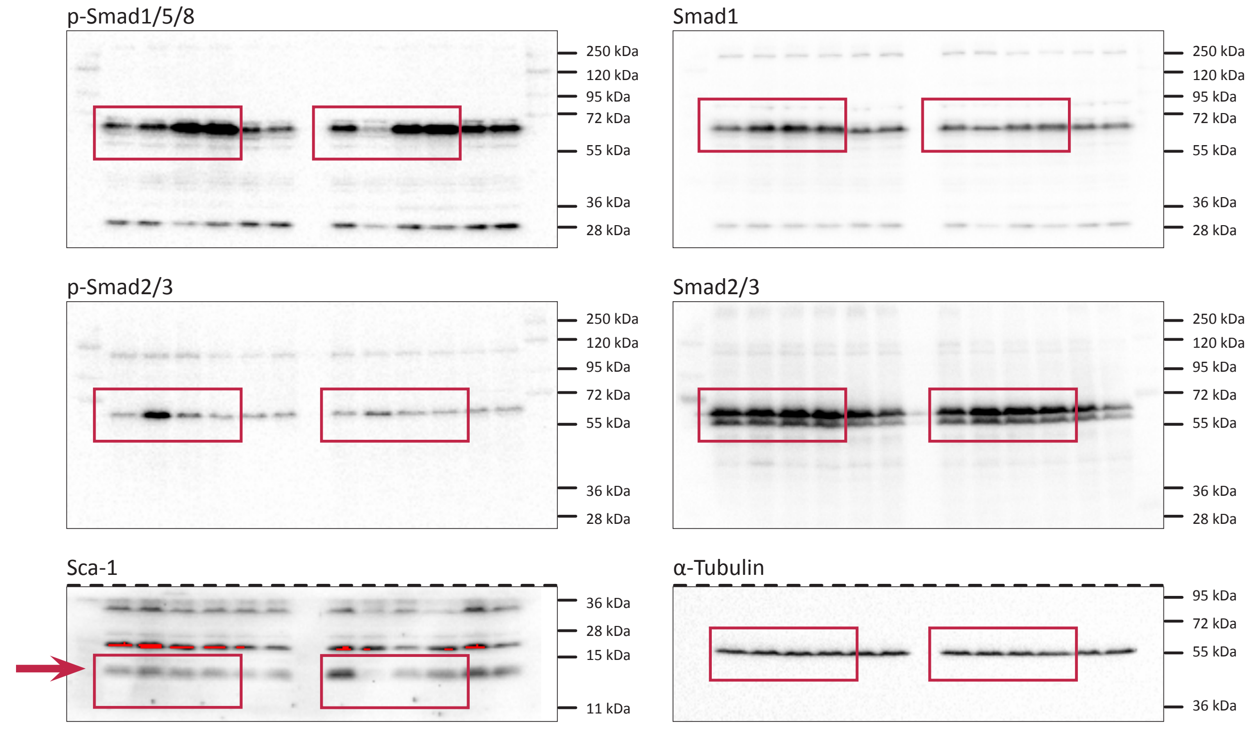

Uncropped membrane scans related to Fig. S6:

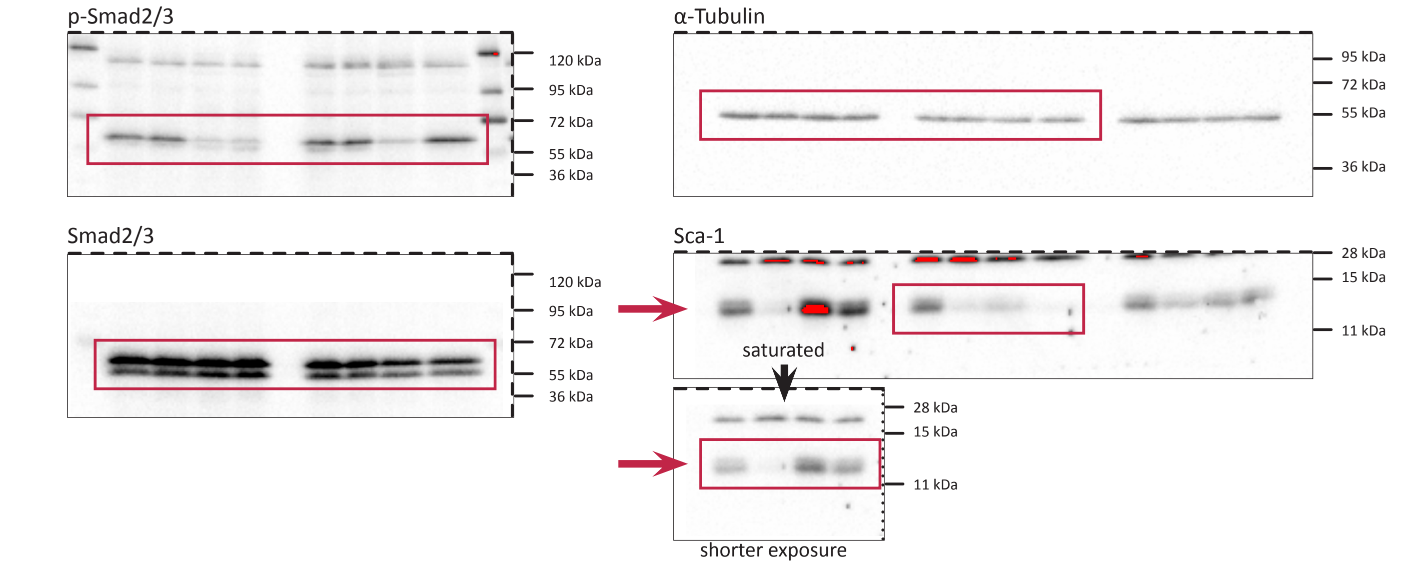

Supplementary Figure 9 - continued

Uncropped membrane scans related to Fig. S3:

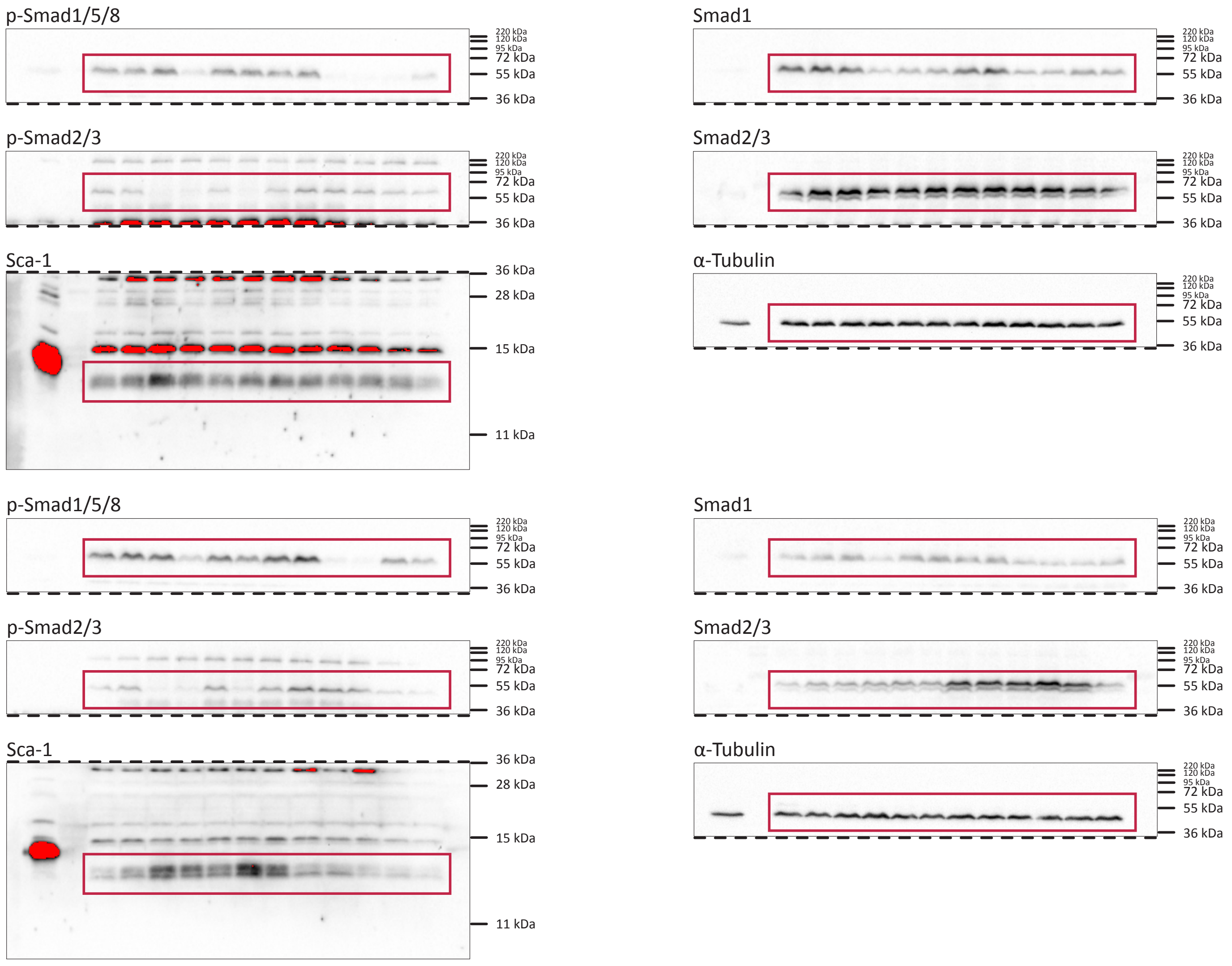

Uncropped membrane scans related to Fig. S6:

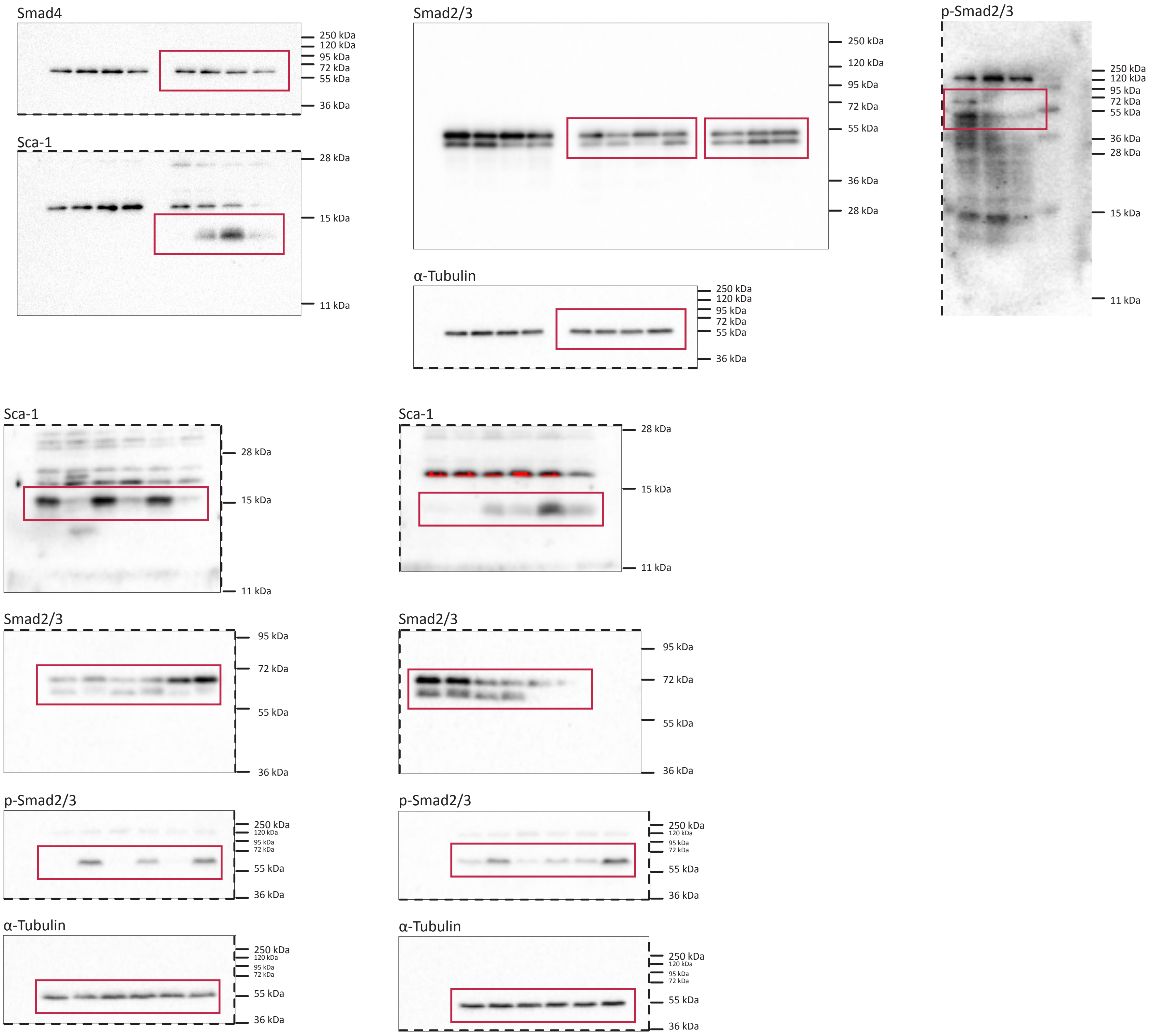

Supplementary Figure 9. Uncropped membrane scans.

Whole blot scans and uncropped membrane scans related to western blots presented in this study.
